# Increasing biomass demand enlarges negative forest nutrient budget areas in wood export regions

**DOI:** 10.1038/s41598-018-22728-5

**Published:** 2018-03-27

**Authors:** Wagner de Oliveira Garcia, Thorben Amann, Jens Hartmann

**Affiliations:** Institute for Geology, Center for Earth System Research and Sustainability, Universität Hamburg, Hamburg, Germany

## Abstract

Energy production from biomass is one of the adopted strategies in different European countries to limit global warming to within the 1.5–2° targets after the 2015 UN climate agreement. This will motivate enhanced forest harvest rates and whole tree harvest to supply the increasing biomass demand. Negative nutrient budgets for certain timberland areas where geogenic nutrient supply cannot cope with harvesting rates will be one consequence. A spatially explicit analysis for a U.S. timberland area of 33,570 km^2^ reveals that for a minimum nutrient loss and supply scenario, negative nutrient budgets occur in 17, 20, 16, and almost 94% of the studied areas for Ca, K, Mg, and P, respectively. For a maximum nutrient loss (considering intensive harvesting) and supply assumptions, the affected areas increase to 50, 57, 45 and 96% for Ca, K, Mg, and P, respectively. In general, atmospheric nutrient deposition is of minor importance for the high weathering supply cases. Increasing global woody biomass demand may cause additional pressure on forested ecosystems, enlarging negative nutrient budget areas. If woody biomass demand rises, strategies to counterbalance nutrient gaps might be needed, for example, by preparing harvested areas with rock products, designed to replenish growth limiting nutrients, and/or implementing forest management strategies to minimize nutrient export.

## Introduction

Global woody biomass use for energy is expected to increase by 2050^1^, driven by the biomass co-firing in conventional coal power plants and household fuelwood^1^ as attempt to decrease net CO_2_ emissions^2,3^. Biomass co-firing might be beneficial in the long run only if the harvested land regrowth reaches the pre-harvest biomass levels, and if the biomass is maintained there^4^. Some authors point out the controversial climate impacts of replacing coal by biomass as an energy source^4^. However, choosing woody biomass for energy production is mainly influenced by its low CO_2_ mitigation costs and its negative financial gap to coal from −0.03 to 0.04 €/kWh_el_^5^ (−8.3 × 10^−9^ to 1.1 × 10^−8^ €/J). EU-27 plus Norway and Switzerland reported a CO_2_ emission reduction by 12.6 × 10^6^ t using wood pellets as an alternative energy source in 2008^6^. Globally retrofitting coal power plants and firing them with 1–10% of biomass is expected to reduce CO_2_ emissions by 45–450 × 10^6^ t per year by 2035^7^.

In 2014, wood and agglomerated wood products, i.e. pellets and briquettes, provided almost half (45%) of EU-28’s total inland energy production by renewables^8^. Current European renewable energy policy will boost woody biomass demand^9^ and, considering 2015 as baseline, the global woody biomass demand is expected to be 23 × 10^6^ t a^−1^ in 2024 representing a 70% increase^10^. For 2050^1^, global woody biomass use for energy is expected to increase by 1.6 × 10^10^ t a^−1^ (obtained from 2.3 × 10^10^ m^3^ a^−1^ by assuming 0.7 t m^−3^ as average woody biomass bulk density) representing a potential energy production ranging from 2.7–3 × 10^20^ J a^−1^ (for a 1.7–1.9 × 10^10^ J t^−1^ biomass’ energy output^11,12^). By the late 21^st^ century, the biomass energy production is expected to be 2.4–8.5 × 10^20^ J a^−1 ^^13^, which is approximately two orders of magnitude higher than the 2016 biomass energy production of 1.8 × 10^18^ J a^−1 ^^14^.

The increasing European demand of forest related biomass requires imports from other areas in the world^15^. Frequent logging residue removal can impact long-term nutrient cycling^16^. Since practices like whole-tree harvest are adopted, the wood and increasingly its “remains” are permanently detracted from the local nutrient cycle. High rates of nutrient export can negatively impact the nutrient budgets in low geogenic nutrient supply areas^16^.

Already, soil nutrient deficiency is observed for forests with intensive harvest practices, e.g. in Germany^17^ and Belgium^18^. Considering tree harvest, negative budgets were reported for North America^19–22^. Deficiency in nutrients causes elevated tree mortality and lower resistance to pests^19^ as well a decrease in biomass productivity^18,23^ and soil fertility^18^. Low tree mineral nutrition is already limiting the biomass yield in European forests^23^.

Natural nutrient pools are divided in short- and medium- to long-term stocks. The short-term nutrient stock in trees, forest floor, and soil has a larger nutrient contribution to tree growth than the long-term stock. The former can be divided in above ground (nutrients in trees and forest floor) and soil nutrients^18,24^. Soil nutrients are expected to be most abundant in the upper 50 cm, while nutrient concentrations decrease with increasing depths^18,25,26^. The medium- to long-term pool is represented by geogenic supply of nutrients from weathering and from atmospheric deposition^24^. In some cases, slow weathering nutrient allocation may limit the biomass yield^20^.

Lateral and partly trans-continental woody biomass exports potentially lead to significant nutrient loss in local ecosystems, which cannot be compensated by geogenic resupply, being itself controlled by local lithology and climatic conditions. This imbalance between harvest nutrient export and geogenic nutrient supply would lead to forest nutritional gaps. However, an evaluation of the potential gap between projected removal rates and the capacity of a system to replenish the geogenic nutrients is necessary. Therefore, exemplary quantification of potential continental United States nutritional gaps is done by quantifying the wood harvesting geogenic nutrient removal and subsequent export for different applied harvesting intensities. The obtained nutrient export is compared to quantified *in-situ* weathering and atmospheric deposition resupply rates. Such a comparison, in principle, enables the local pools potential nutrient depletion prediction for different harvesting rates and reforestation scenarios. Predicting potential nutrient depletion may help to guide future forest management practices^24^. The objective here is to evaluate if geogenic nutrient supply is able to meet forest nutrient demand under high harvest rates for an increasing bioenergy demand in the future.

## Methods

### Timberland wood composition and nutrient loss

Different variables control the nutrient concentration within biomass compartments, resulting in high nutrient variability in trees^27^, which only enables first order large scale estimates. Based on the U.S. forest type distribution map^28^ and a tree chemistry database^29^, the lateral exports for Mg, Ca, K, and P nutrients by wood harvest was quantified.

Considering future bioenergy demand increase, a complete dead wood, stem, bole, branch, twig, and foliage harvest is assumed^18,30^, making it possible to neglect the nutrient contribution by *in-situ* biomass decay. In addition, a scenario is provided assuming twigs and leaves remain in the ecosystem. Wood harvest area distribution and harvest intensities (Supplementary Information (SI) section A), ranging from ≤140 to ≥1574 m^3^ km^−2^, were taken from the U.S Forest Service^31^ (SI Fig. S1). Mg, Ca, K and P loss rate of ecosystems based on these harvest rate intensities were calculated:

1 $$Nutrient\,loss\,({N}_{l})={M}_{i}\cdot {C}_{w}$$

with

2 $$Wood\,yield\,({M}_{i})=Clas{s}_{i}\cdot {\rho }_{wood}\cdot {V}_{b}$$

where ${N}_{l}$ represents the nutrient loss [kg km^−2^ a^−1^] calculated for 25^th^ or 75^th^ exported nutrient quartiles, *M*_*i*_ is the area normalized wood harvested mass [kg km^−2^ a^−1^], *C*_*w*_ [−] is the 25^th^ or 75^th^ quartile fraction of each nutrient *w* within Timberland wood (SI Table S1), *Class*_*i*_ [m^3^ km^−2^] represents the minimum or maximum harvest rate per harvest class provided by the U.S Forest Service^31^, ρ_*wood*_ [kg m^−3^] is the wood density^32^ (SI Table S1), ${V}_{b}$ is a correction factor for bundled wood volume, depending on material properties such as tortuosity, homogeneity, diameter and log length, assumed to be 0.7 [−]^33^.

### Nutrient supply

Nutrients are sourced from weathering and atmospheric deposition. They consider spatially explicit and averaged data. Geogenic nutrient supply is the sum of weathering nutrient fluxes and atmospheric nutrient precipitation. Total (wet + dry) atmospheric nutrient precipitation rates from atmospheric deposition maps^34^ were used for obtaining the applied 25^th^/75^th^ quartiles and median deposition rates for Mg, Ca, and K for 2000 until 2015 (SI section B1, Figs S3 to S5). The phosphorus atmospheric deposition rate was obtained from a global model^35^ with a coarser resolution than for the other elements (SI section B1 and Fig. S5).

Nutrient supply from chemical weathering for twelve aggregated lithological classes (SI section B3) are estimated assuming complete mass dissolution, based on spatially-explicitly modelled weathering rates from literature (SI section B2). To assess the probable long-term nutrient release range, the 25^th^/75^th^ quartiles and median geochemical compositions for each lithological class were derived from geochemical databases (SI Table S4). The overall nutrient release rate (SI section B4) is then calculated assuming a nutrient release rate proportional to the nutrient content in the lithological class relative to the sum of base cations released (Mg, K, Ca, Na, and Si):

3 $$N{f}_{calc}=W{R}_{calc}.\frac{{C}_{e}}{{\sum }_{i}^{n}.{C}_{i}}$$

where ${{Nf}}_{{calc}}$ is the total nutrient release rate via weathering to the soil-ecosystem for *e*lement *e* [kg km^−2^ a^−1^], which is Mg, K, Ca, or P. *WR*_*calc*_ is the weathering rate [kg km^−2^ a^−1^] taken from the model output after Hartmann *et al*.^36^ (SI section B2). *C*_*e*_ is th*e* element *e* (Mg, K, Ca, or P) concentration. *C*_*i*_ is the sum of base cations and silicon released via weathering concentration [weight-%]. For each considered element within a lithological class, the 25^th^ and 75^th^ percentiles were used as minimum and maximum boundary scenarios (SI section B3 and Table S4). Spatially explicit weathering nutrient release from each lithological class were used for quantifying the 25^th^/75^th^ quartiles and median weathering nutrient fluxes (SI section B4), which were added to the atmospheric precipitation rates to quantify the total geogenic nutrient fluxes (SI section B5).

The averaged geochemical nutrient fluxes were compared to river hydrochemical fluxes from U.S. watersheds covered with at least 95% of forests. The comparison should provide an estimate of the considered geogenic nutrients leaching (SI section B5 Fig. S8). As dissolved compounds’ leaching is in general lower than calculated geogenic supply, the deficit calculations presented are interpreted as being conservative.

### Nutrient budget

The spatially-explicit nutrient budget for geogenic nutrient supply and nutrient export was done to evaluate the actual system’s nutrient situation. The spatially-explicit nutrient budget considers geogenic nutrient supply and nutrient loss by practiced harvest rates derived from spatially-explicit information. The procedures for obtaining the spatially-explicit information for geogenic nutrient supply and harvest loss is described in SI section C. The resulting maps for each element are presented in the same SI section (Figs S18 to S25).

**Figure 1:**
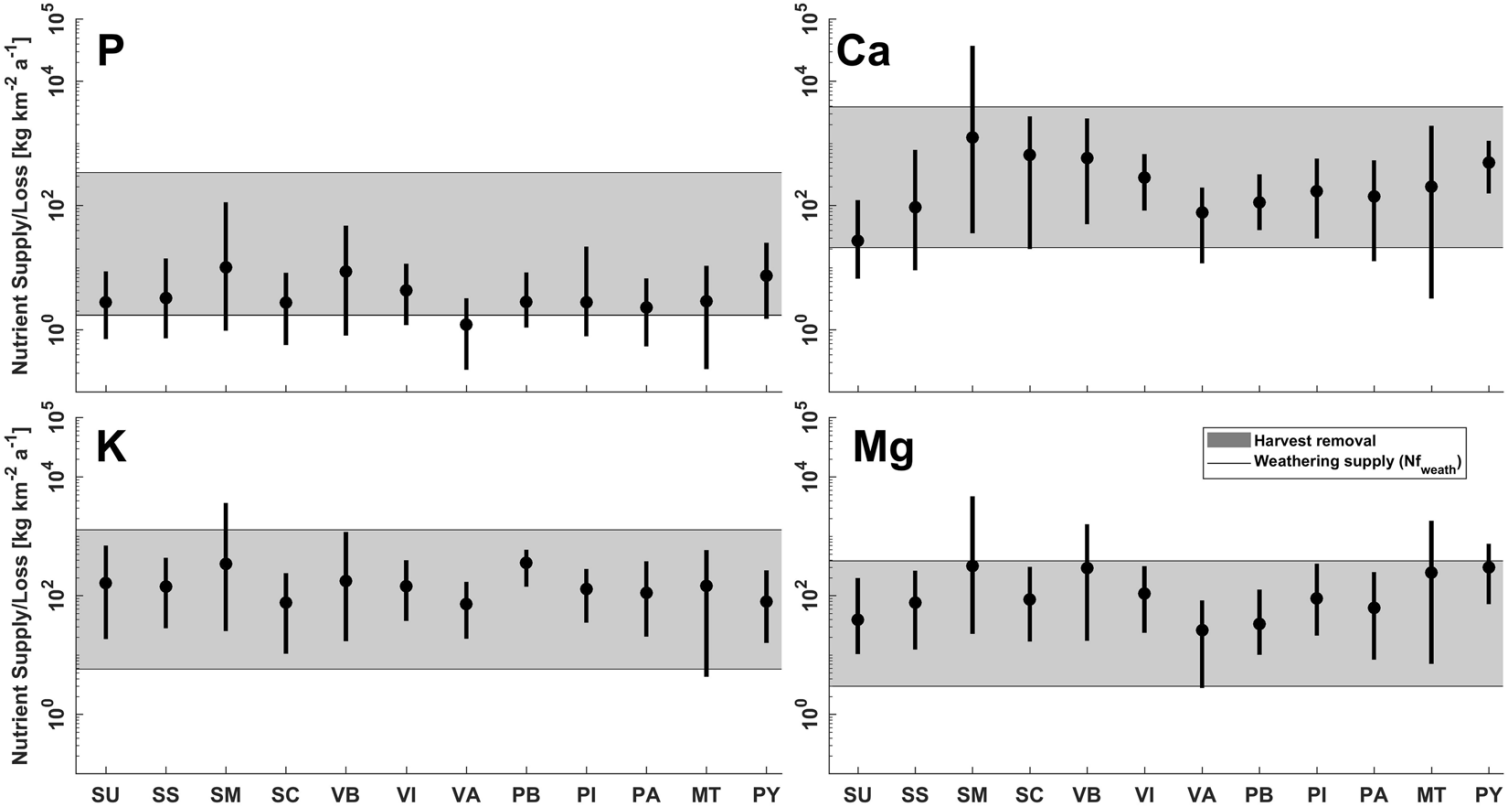
Weathering nutrient supply averaged for all spatially explicitly studied areas, considering median (filled circles), minima, and maxima (whiskers) nutrient supply, compared to the potential nutrient loss by clear-cut scenarios (horizontal grey filled boxes). Harvest rates ranging between 70 m^3^ km^−2^ a^−1^ (Scenario 1 from SI Fig. S17 section C) and 3150 m^3^ km^−2^ a^−1^ (Scenario 8 from SI Fig. S17 section C). Nutrient loss for scenarios 2 to 7 from SI Fig. S17 correspond to the shaded areas. Abbreviations: Unconsolidated sediments (SU), siliciclastic sedimentary rocks (SS), mixed sedimentary rocks (SM) and carbonate sedimentary rocks (SC) representing the group of sedimentary rocks. Basic volcanic rocks (VB), intermediate volcanic rocks (VI) and acid volcanic rocks (VA), represent the volcanic rock group. Basic plutonic rocks (PB), intermediate plutonic rocks (PI) and acid plutonic rocks (PA), constitute the plutonic rock group. Metamorphic rocks (MT) and pyroclastic rocks (PY). The number of samples used for rock class composition statistics (n values) are presented in SI Table S4.

**Figure 2:**
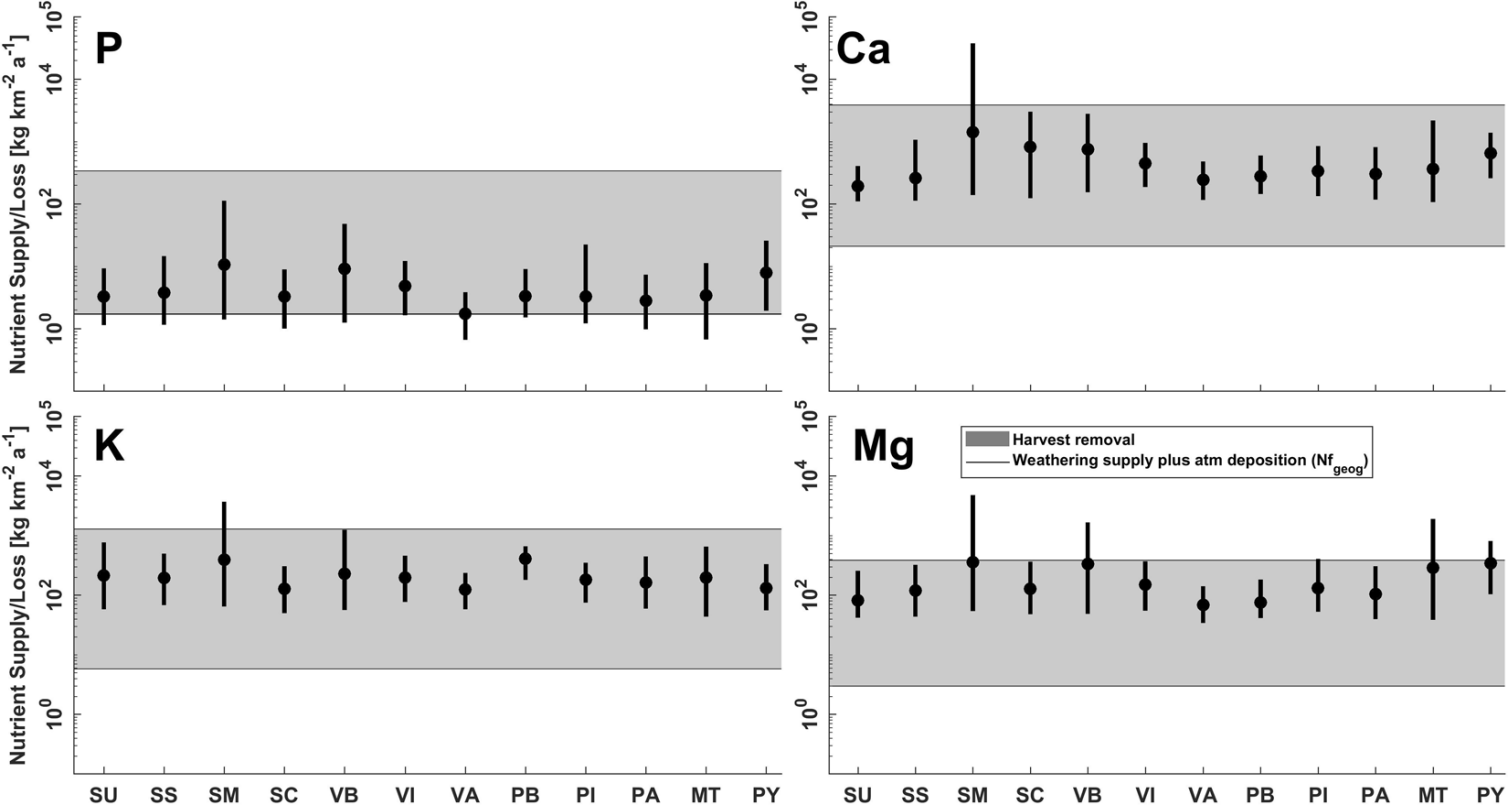
Total assumed geogenic supply by weathering and atmospheric deposition averaged for all spatially explicitly studied areas, considering median (filled circles), minima, and maxima (whiskers) nutrient supply, compared to the potential nutrient loss by clear-cut scenarios (horizontal grey filled boxes). Harvest rates ranging between 70 m^3^ km^−2^ a^−1^ (Scenario 1 from SI Fig. S17 section C) and 3150 m^3^ km^−2^ a^−1^ (Scenario 8 from SI Fig. S17 section C). Nutrient loss for scenarios 2 to 7 from SI Fig. S17 correspond to the shaded areas. For abbreviations refer to Fig. 1.

Diagrams to predict nutrient supply efficiency for different harvest nutrient loss scenarios considered eight differentiated scenarios (SI section C Fig. S17). Special attention is given to scenarios 1 and 8, as they represent the overall 25^th^ and 75^th^ percentiles for nutrient supply and nutrient harvest losses. For harvest loss, scenarios 1 and 8 are represented by the inferior and superior horizontal limits of the grey boxes in Figs 1 and 2. The scenarios 2 to 7 correspond to the filled grey boxes. For the studied timberland area weathering supply only (Fig. 1) and total geogenic supply, including atmospheric deposition (Fig. 2), the vertical lines’ lower and upper limits correspond to the 25^th^ and 75^th^ nutrient supply percentiles, while the filled circles represents the median values. The diagrams allow for a general discussion and provide an easy to understand tool to rapidly identify the potential weathering (Fig. 1) or geogenic (Fig. 2) general nutrient balance for a chosen harvest rate. The detailed spatially explicit nutrient budgets calculations are shown in SI section C for each element.

## Results and Discussion

Nutrient supplies and losses for the total studied area, considering either supply by weathering, or weathering plus atmospheric deposition, are presented distinguishing supply scenarios for the given lithological classes (Figs 1 and 2). Differences in weathering supply rates can be related to the spatial correlation between the lithological geochemical composition, climate and weatherability of the lithological class, which underlines the relevance of lithology for the calculated budgets provided in the SI. For the presented data (Figs 1 and 2), the harvest rate related nutrient loss is constant, while the nutrient supply rates are variable.

Averaged nutrient loss and given weathering supply scenarios (Fig. 1) suggest that in general the phosphorus supply, for all lithological classes, cannot support the highest reported harvest rate of 3150 m^3^ km^−2^ a^−1^. For other nutrients, the highest losses can only be countered by certain lithological classes, depending on the element. However, in a spatially explicit case and considering maximum reported harvest rates, this might be different depending on the locality, as discussed below. For the lowest considered harvest rate of 70 m^3^ km^−2^ a^−1^, which is unlikely to occur in an intensive bioenergy demand scenario, averaged nutrient export potentially does not exceed the weathering supply for all investigated nutrients and for all lithological classes, with exception of one case (Fig. 1). If additional atmospheric nutrient deposition is taken into account, differences between nutrient supply and loss would decrease, depending on the harvest rate (Fig. 2).

Comparing the geogenic nutrient supply values from Fig. 2 to the measured averaged weathering/leaching rates, based on stream water samples of U.S. catchments covered with at least 95% forests, suggests overestimation for Mg, Ca, K, and P by two orders of magnitude or more (SI section B5 Fig. S8). However, physical erosion is another relevant nutrient loss term that is not considered here, and would demand further evaluation for forested areas to address certain erosion caused sinks. This aspect remains a critical sink term to be investigated for timberland area, which would show elevated physical erosion, in comparison to natural forests^37–39^.

Atmospheric Ca, K, Mg, and P deposition can be locally important for nutrient supply if weathering nutrient supply is low, like in dry areas. To highlight this, maps plotting the difference by subtracting the weathering supply from the atmospheric deposition supply were calculated for different supply scenarios (SI Figs S9 to S16). In general, the atmospheric deposition plays a minor role for the considered timberland areas, but can locally be relevant (cf. SI section B5).

Local forest management would need regional data to reliably adjust the nutrient resupply to losses. Spatially explicit results for the studied 33,570 km^2^ U.S. timberland area suggest that harvest nutrient loss exceeds geogenic nutrient supply for a significant proportion of that area, given the continental scale analysis approach. Considering a conservative scenario with minimum harvest nutrient loss and geogenic nutrient supply, negative budgets exist for Ca, K, Mg, and P in 17, 20, 16, and 94% of the timberland area, respectively (SI section C and Figs S18 to S21). For a maximum harvest nutrient loss and geogenic nutrient supply, the affected areas with a negative budget increase to 50, 57, 45, and 96% for Ca, K, Mg, and P, respectively (SI section C and Figs S22 to S25).

Higher harvest rotation frequencies are expected to meet an increasing biomass demand for energy production^1,10,13^. Rotations and tree clear-cut intensification will widen the areas with a negative nutrient budget. To manage these gaps between nutrient supply and loss, and to avoid growth limitations, a sustainable forest management will rely on external nutrient sources to provide a long-term balanced system. However, to assess when a system becomes growth limited by shortage of one or more of the nutrients discussed is still a matter of debate^18,23^.

Aside from negative nutrient budget issues, the wood harvest intensification may increase soil nutrient leaching^40–42^, runoff and soil erosion rates^37–39^, and the organic carbon loss from soils if no countermeasures are taken^43–47^.

Whole-tree or clear-cut harvests magnify nutrient losses due to biomass export. From the analysis, harvest rates and nutrient export are proportionally related. Implementing lower harvest rates would diminish nutrient export, decrease nutritional gaps and, in some cases, even avoid them or lead to a positive nutrient budget. Logging residue removal can negatively impact the long term nutrient balance^16^, especially in low geogenic nutrient resupply regions. An alternative practice to keep the long term nutrient balance is to leave the logging residues (branches and tops) on the harvested site due to their high nutrients concentration relative to other tree compartments^48^.

For the spatially explicit data, if harvest remains are left in the field, calculated negative budget areas decrease only slightly to 16, 17, 15, and 93% of the total area for Ca, K, Mg, and P respectively, for a conservative scenario with minimum harvest nutrient loss and geogenic nutrient supply. For a maximum harvest nutrient loss and geogenic nutrient supply negative budget areas would decrease to 46, 51, 42, and 95% of the total area for Ca, K, Mg, and P respectively. Therefore, this practice to restore a balanced nutrient budget does not seem to be suitable for all locations.

Suitable rock products as slow-release nutrient sources (on decennial timescales) are an alternative that might be used to artificially replenish the system^49^ for harvest rotations in a centennial time span. Mafic or carbonate rock sources may be suitable for Ca and Mg supply, while more felsic plutonic rock sources^50^ might be needed to supply K. Excess cation release, not taken up by plants, has the potential to sequester atmospheric CO_2_^49,51,52^. Coupling the application of rock products with other soil amendment strategies, such as biochar, may increase the plant nutrient availability by increasing soil’s cation exchange capacity, especially from highly weathered soils of low fertility^53^.

Some of the exported nutrients can potentially be returned to the catchment by the application of ash residues from bioenergy production^54^, and probably mitigate nutrient loss. This practice can also supply nutrients like Ca, Mg, K^54,55^, and P^56,57^, without extra N input^17^ at the same time, therefore, creating a reuse of ash remnants from biomass burning (evaluating the nutrient cycle of the considered elements). However, in this case, issues of solubility speed and nutrient release from ash must be addressed^54^ to prevent early loss from the system via lateral water transfer to river systems. In 2014, European countries imported 20.5 × 10^6^ t of wood pellets^58^ representing an inlet of 3.7, 24.6, 12.1 and 1.8 × 10^3^ t of Mg, Ca, K, and P, respectively. For K and P, it represents 0.5 and 0.3% of 2014 western and central Europe fertilizer consumption^59^.

## Conclusions

Tree nutrient removal by high harvest rates, within studied timberland areas, can often not be compensated by atmospheric deposition and weathering nutrient supply. Increasing future woody biomass demand will likely lead to intensified forest harvesting. Growing rates for reaching the demand may be restrained by negative nutrient budgets due to limiting kinetics in geogenic nutrient resupply^36^. Additionally, high harvest rates will trigger enhanced soil nutrient leaching^40–42^, runoff, and soil erosion rates^37–39^, decreasing the nutrient stocks. It has been experimentally shown that an expected fertilization effect of increasing atmospheric CO_2_ can be potentially prevented by limited nutrient stocks^60^. However, the numbers of studies focusing on this effect or nutrient limitations in biomass production for future bioenergy demands are lacking. Therefore, the additional biomass amount, which can be produced by closing the supply-demand gap, is until now not known. Compilations of studies, which provide the needed parameters to optimize forest biomass production for a given climate, lithological underground, soil and atmospheric deposition would guide and assist future large or global scale forest management strategies.

Negative nutrient budgets can be avoided by decreasing harvest intensities, recycling harvest remains^48,61^ and/or by providing an external nutrient input, either by industrial agrochemicals or natural rock products of specifically tailored geochemical character^49,52,62^. However, proper knowledge on spatially explicit limitations on forest biomass growth rates is still missing, yet would be needed to assess a realistic global forest bioenergy potential and to close local geogenic nutrient gaps by appropriate measures^17,18,23,42^.

Through the export of wood products, nutrients are transported across continents, e.g. nutrients taken from North America are exported to Europe. Remains from biomass combustion represent a yet mostly untapped source of nutrients which could partly buffer increasing nutrient deficiencies, if they re-enter the local scale nutrient cycles, and if early flushing out of the system can be avoided^54^.

This study presents an overview for timberland nutrient budgets, considering increasing bioenergy demand. Empirical data is necessary to assess and verify global effects of projected increasing harvest rates. Therefore a multitude of tailored local scale studies and compilations of past studies might be necessary. The development of proper weathering models to calculate nutrient budgets for local forest management is necessary, too. Details on nutrient requirements and geogenic nutrient supply would allow location-specific cataloguing of the geogenic nutrient demand for reforestation procedures based on lithologic, climatic and soil properties. As natural geogenic supply will probably not be able to cope with increasing biomass demands, forest management alternatives for long-term nutrient resupply are needed.

## Electronic supplementary material

Supplementary information

Database 1
