# Supplementary material for: Increasing biomass demand enlarges negative forest nutrient budget areas in wood export regions
Source: Sci Rep. 2018 Mar 27;8:5280. doi: 10.1038/s41598-018-22728-5 (PMC5869705; doi:10.1038/s41598-018-22728-5)
Supplement: Supplementary file 1 — Supplementary information [file 41598_2018_22728_MOESM1_ESM.pdf]

Increasing biomass demand enlarges negative forest nutrient budget areas in wood export regions

Authors: Wagner de Oliveira Garcia*, Thorben Amann, Jens Hartmann

Affiliation: Institute for Geology, Center for Earth System Research and Sustainability, Universität Hamburg, Germany

*e-mail: wagner.oliveira.garcia@uni-hamburg.de or wagner.o.garcia@gmail.com

Supplementary information

A. Timberland wood composition and nutrient loss 2

B. Nutrient Supply 12

C. Nutrient budget 28

D. References 33

Abbreviation

N_l	nutrient loss	[kg km^{-2} a^{-1}]
M_i	harvested wood area normalized mass	[kg km^{-2} a^{-1}]
C_w	minimum or maximum nutrient 'w' fraction within exported material	[-]
$Class_i$	harvest rates	[m^3 km^{-2}]
ρ_{wood}	wood density	[kg m^{-3}]
V_b	wood packed volume correction	[-]
Nf_{calc}	total nutrient release to the soil-ecosystem*	[kg km^{-2} a^{-1}]
WR_{calc}	weathering rate	[kg km^{-2} a^{-1}]
C_i	cations and silicon lithological class concentration	[weight-%]
C_e	element 'e' lithological class concentration	[weight-%]
tf	tree compartment fraction	[-]
tcc	nutrient 'w' tree compartment chemistry	[weight-%]

*Nf_{calc} was split in weathering only (Nf_{weath}) and in geogenic (weathering plus atm. deposition) nutrient flux (Nf_{geog}) to understand the different sources contribution.

A. Timberland wood composition and nutrient loss

Spatially-explicit nutrient budget for whole-tree harvest/clear-cut was investigated. The whole-tree nutrient contribution was accounted by the nutrient content sum within: dead wood, stem, bole, branch, twig, and foliage based on each compartment fraction. The spatially averaged nutrient loss and supply diagrams are presented in the main text and are used for estimating specific harvest rates nutritional requirements (no spatial distribution considered) by visualizing the harvest rate nutrient losses and the possible geogenic (weathering plus atmospheric deposition) nutrient supply from different lithological classes.

Detailed calculation procedures for spatially-explicit nutrient budgets and spatially averaged diagrams are described in the sections A) the nutrient loss, B) the nutrient supply, and C) presenting the spatially-explicit analysis for deficits per considered nutrient, in addition to the diagrams presented in the main text.

A. Timberland wood composition and nutrient loss

Spatially-explicit information for timberland area and harvest rates were obtained from a U.S. Forest Service[1] shapefile (Fig. S 1a). The spatially-explicit harvest rates are divided in seven classes with minima and maxima harvest rate values according to U.S. Forest Service[1] as follow: <140, 140 – 343, 350 – 588, 595 – 833, 840 – 1148, 1154 – 1571 and >1574 m^3 km^{-2}. One considered group for analysis represents minimal harvest rates per class with: 70 (the minimum reported value half), 140, 350, 595, 840, 1155 and 1575 m^3 km^{-2}. The second group represents the maximal harvest rates per class: 139 (for <140), 343, 588, 833, 1148, 1567 and 3150 m^3 km^{-2} (with 3150 m^3 km^{-2} being 2 times higher than 1574 m^3 km^{-2}, the reported upper boundary). The practiced harvest rates for minimum and maximum nutrient loss were used in $Class_i$ from equation 2 in the main text for quantifying the spatially-explicit nutrient loss and for diagrams representing harvest rate averaged nutrient loss (Fig. S 1b).

To assess the spatially-explicit tree-species within timberland areas, the timberland distribution shapefile was merged with the forest cover type raster-file from the U.S. Forest Service and U.S. Geological Survey[2]. The 22 tree-species identified within the mapped timberland area had the chemical composition derived from the Tree Chemistry Database[3]. The green wood density was derived from the U.S. Forest Products Laboratory[4] for each of 22 tree-species. In some localities, more than one tree-species was described within an area, which originates a composed tree-species name (e.g. Fir-Spruce). In those cases, all data concerning the different tree-species were used to calculate the 25th/75th quartiles and median values for tree chemistry and density. When a tree-species could not be identified within the chemistry or density databases, values assuming the 25th/75th quartiles and median of all trees in the databases were used (Fig. S 2). This occurred for the Chaparral, Douglas-fir, Elm-ash-cottonwood, Hemlock-Sitka spruce, Larch, Oak-hickory, Redwood, Loblolly-shortleaf-pine and Pinyon-juniper entries for chemistry and Pinyon-juniper entries for density (Table S 1).

A. Timberland wood composition and nutrient loss

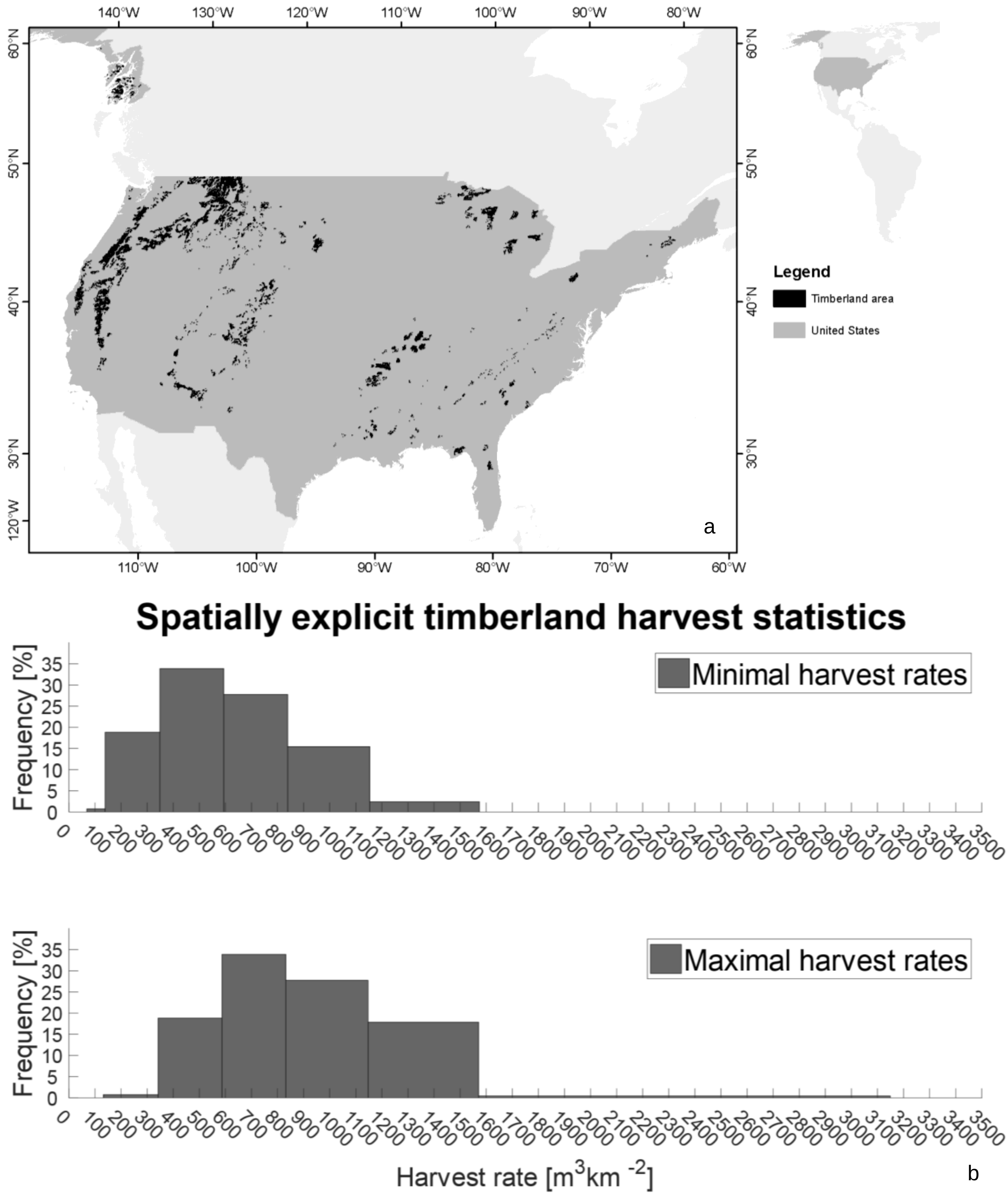

Figure S 1: a) Timberland distribution area[1] across the continental U.S. (total area: 37,536 km^2). Pixel outline width border of 0.01. Map generated with ESRI ArcGIS ver. 10.3.1 (http://www.esri.com). b) Minimal and maximal harvest rate frequencies considering U.S. Forest Service[1] explicit data. Within the minimal harvest rate group, the most frequent harvest rates are 350 and 595 m^3 km^{-2}. For the maximum harvest rate group, 588 and 833 m^3 km^{-2} are the most frequent harvest rates.

A. Timberland wood composition and nutrient loss

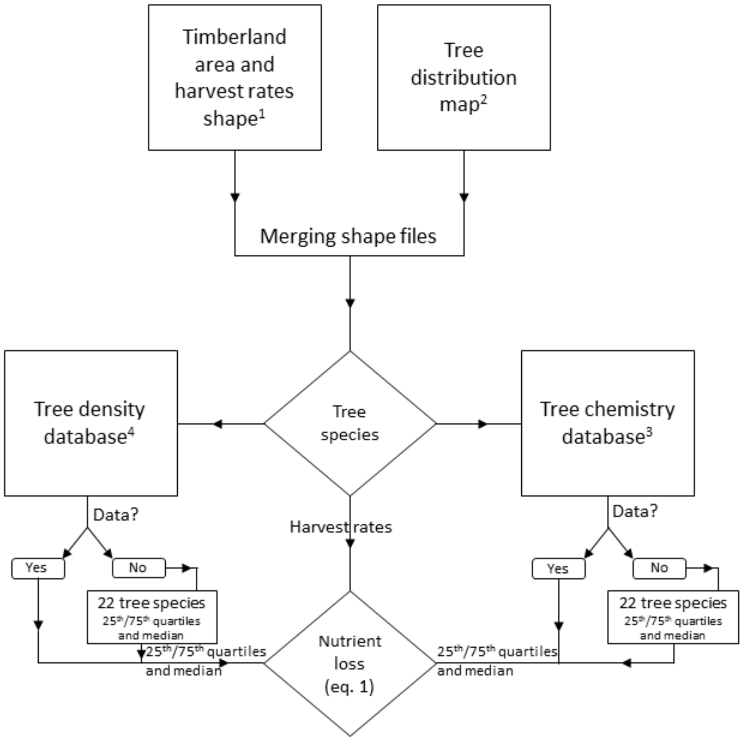

Figure S 2: Workflow for obtaining the spatially-explicit 25th/75th quartiles and median nutrient loss within timberland areas by using spatially-explicit harvest rates, whole tree-species chemistry and density database for green wood. For the spatially averaged diagrams, the previous steps where followed, but instead of using spatially-explicit harvest rates, the values within minimal and maximal harvest rate groups were used for each one of the 22 tree-species. The final values were averaged and used on the diagrams.

A1) Compartment weighted chemical composition per considered tree-species

Information concerning the chemistry and mass fraction for the following tree compartments were available: dead wood, stem, bole, branch, twig, and foliage. For the considered timberland clear-cut scenarios, the whole-tree chemical composition was estimated by:

(SI-1)
$$C_w = perct(\frac{\sum(tf * tcc)}{100})$$

Where C_w is the exported whole-tree chemistry fraction (Table S 1) for the $25^{th}/75^{th}$ quartiles and median of each nutrient w [-] within the chosen tree-species. The $25^{th}/75^{th}$ quartiles and median values were obtained considering all the tree compartments listed before (Table S 2) for 'n' samples (Table S 1). C_w is used in main text equation 1. tcc [weight-%] is the tree compartment chemistry of nutrient w (e.g. twig, etc.) and tf [-] (Table S 3) is the tree compartment fraction (e.g. twig, etc.). tcc and tf were derived from tree chemistry database[3].

A. Timberland wood composition and nutrient loss

A2) Estimating the spatially-explicit element loss and applied minima and maxima scenarios

Minima and maxima spatially-explicit nutrient loss N_l (equation 1 main text) using harvested biomass area normalized mass M_i (equation 2 main text) was estimated by using the following parameter settings affecting the elements' export rate:

1) The 25th/75th quartiles from the spatially-explicit exported whole nutrient fraction C_w [-] (SI-1) per tree-species and considered element are used here as respectively minima and maxima values for the discussion (cf. Table S *1*).

2) Minima and maxima values of each spatially-explicit harvest rate class $Class_i$ [m^3 km^{-2}] named above (cf. First paragraph section A).

3) The 25th/75th quartiles from wood density ρ_{wood} [kg m^{-3}] per tree-species used as minima and maxima case values for the discussion (cf. Table S *1*).

Sometimes a mismatching between geospatial information sources was observed. Areas were marked as non-forest within the forest type map[2] despite of mapped presence of timberland by the U.S. Forest Service[1]. In this case, the 25th/75th quartiles and median values for whole-tree chemical composition and green wood density of all the 22 considered tree-species were assumed, which were then multiplied by the minimum or maximum spatially-explicit harvest rates reported for the mapped timberland area.

A3) Estimating the nutrient loss for different harvest rates

Like the spatially-explicit nutrient loss, the nutrient loss for a harvest rate was calculated by main text equation 1 and 2. Minimal and maximal harvest rate groups' values (cf. first paragraph section A) were used as harvest rate class ($Class_i$ in main text equation 2), allowing for an analysis that does not consider spatially-explicit data. The reported 25th/75th quartiles and median green wood density values of each tree-species within timberland area were used as ρ_{wood} (Table S 1). The 25th/75th quartiles and median values for whole-tree chemical compound fraction for each tree-species were used as C_w (Table S 1). Minima and maxima values for nutrient loss were obtained for all 22-tree-species. The nutrient losses of each harvest rate and tree-species were grouped together and averaged. The resulting value was used for nutrient loss prediction. In the main text Fig.1 and Fig. 2, harvest rate nutrient losses are presented by the grey horizontal boxes.

A. Timberland wood composition and nutrient loss

Table S 1: Exported nutrient content statistics for each tree-specie for clear-cut obtained by equation SI-1, considering the nutrient content within dead wood, stem, bole, branch, twig, and foliage. Wood density for 25th, 75th quartiles and median of each considered tree-specie.

	Ca[3]				Mg[3]				K[3]				P[3]				Density ρ_{wood}[4]					
Unit	[WT%]																[kg m^{-3}]					
Tree specie	25th	med	75th	n	25th	Med	75th	n	25th	med	75th	N	25th	med	75th	n	25th	med	75th	N	n_tf	n_tcc
Aspen-birch	0.22	0.30	2.02	10	0.03	0.04	0.07	10	0.08	0.14	0.20	10	0.03	0.03	0.03	10	424	453	474	5	10	10
Chaparral*	0.20	0.28	0.35	26	0.02	0.03	0.04	26	0.06	0.09	0.12	26	0.01	0.03	0.03	26	460	460	460	1	0	0
Douglas-fir*	0.20	0.28	0.35	26	0.02	0.03	0.04	26	0.06	0.09	0.12	26	0.01	0.03	0.03	26	450	455	460	4	0	0
Elm-ash-cottonwood*	0.20	0.28	0.35	26	0.02	0.03	0.04	26	0.06	0.09	0.12	26	0.01	0.03	0.03	26	421	440	479	11	0	0
Fir-spruce	0.15	0.27	0.32	9	0.02	0.03	0.03	9	0.05	0.09	0.11	9	0.01	0.03	0.04	9	333	365	371	12	9	9
Hemlock-Sitka spruce*	0.20	0.28	0.35	26	0.02	0.03	0.04	26	0.06	0.09	0.12	26	0.01	0.03	0.03	26	370	403	404	11	0	0
Larch*	0.20	0.28	0.35	26	0.02	0.03	0.04	26	0.06	0.09	0.12	26	0.01	0.03	0.03	26	480	480	480	1	0	0
Loblolly-shortleaf pine*	0.20	0.28	0.35	26	0.02	0.03	0.04	26	0.06	0.09	0.12	26	0.01	0.03	0.03	26	380	430	470	32	0	0
Lodgepole pine	0.09	0.23	0.37	4	0.02	0.02	0.03	4	0.03	0.05	0.06	4	0.01	0.02	0.03	4	380	430	470	16	4	4
Longleaf-slash pine	0.09	0.23	0.37	4	0.02	0.02	0.03	4	0.03	0.05	0.06	4	0.01	0.02	0.03	4	380	430	470	32	4	4
Maple-beech-birch	0.20	0.22	0.28	7	0.02	0.03	0.03	7	0.06	0.09	0.11	7	0.01	0.02	0.03	7	499	533	559	9	7	7
Oak-gum-cypress	0.27	0.27	0.27	1	0.03	0.03	0.03	1	0.09	0.09	0.09	1	0.03	0.03	0.03	1	490	500	510	18	1	1
Oak-hickory*	0.20	0.28	0.35	26	0.02	0.03	0.04	26	0.06	0.09	0.12	26	0.01	0.03	0.03	26	580	598	620	25	0	0
Oak-pine	0.09	0.23	0.37	4	0.02	0.02	0.03	4	0.03	0.05	0.06	4	0.01	0.02	0.03	4	470	505	535	33	4	4
Pinyon-juniper*	0.20	0.28	0.35	26	0.02	0.03	0.04	26	0.06	0.09	0.12	26	0.01	0.03	0.03	26	380	460	560	117	0	0
Ponderosa pine	0.09	0.23	0.37	4	0.02	0.02	0.03	4	0.03	0.05	0.06	4	0.01	0.02	0.03	4	380	430	470	16	4	4
Redwood*	0.20	0.28	0.35	26	0.02	0.03	0.04	26	0.06	0.09	0.12	26	0.01	0.03	0.03	26	350	380	440	3	0	0
Spruce-fir	0.15	0.27	0.32	9	0.02	0.03	0.03	9	0.05	0.09	0.11	9	0.01	0.03	0.04	9	333	365	371	12	9	9
Western hardwoods	0.09	0.23	0.37	4	0.02	0.02	0.03	4	0.03	0.05	0.06	4	0.01	0.02	0.03	4	427	435	443	20	4	4
Western white pine	0.09	0.23	0.37	4	0.02	0.02	0.03	4	0.03	0.05	0.06	4	0.01	0.02	0.03	4	380	430	470	16	4	4
White-red-jack pine	0.17	0.35	0.39	3	0.02	0.03	0.03	3	0.03	0.06	0.06	3	0.01	0.03	0.03	3	380	430	470	48	3	3
Alaska Spruce-birch	0.21	0.26	0.29	10	0.02	0.03	0.03	10	0.06	0.09	0.10	10	0.03	0.03	0.03	10	414	460	480	8	10	10

n is the number of samples used in the statistic. The values for Ca, Mg, K, and P correspond to C_w in main text equation 1. For areas with more than one tree-species $25^{th}/75^{th}$ and median values were used as C_w. *Trees without tree compartment fraction information, in those cases, average C_w values were used. n_tf and n_tcc correspond to the number of trees with tree compartment fraction information and tree compartment chemistry respectively.

A. Timberland wood composition and nutrient loss

Table S 2: Whole tree chemistry. Values obtained considering the nutrient content and fraction of different tree compartments (equation SI-01). Each row represents the calculated fraction of different trees using the Tree Chemistry Database[3]. The raw table is given in the excel supplement.

Tree specie	Ca	Mg	K	P
Unit	[-]			
Aspen-birch	2.6E-03	3.8E-04	8.9E-04	3.0E-04
	1.7E-03	1.9E-04	4.0E-04	9.8E-05
	2.2E-03	2.8E-04	6.4E-04	2.7E-04
	2.1E-03	2.3E-04	1.1E-03	2.7E-04
	3.4E-03	4.7E-04	1.8E-03	3.2E-04
	2.7E-03	3.0E-04	8.2E-04	1.4E-04
	5.2E-02	5.8E-04	2.0E-03	3.2E-04
	3.6E-02	8.7E-04	7.1E-03	4.8E-04
	2.0E-02	7.2E-04	1.9E-03	3.5E-04
	3.7E-03	6.7E-04	2.2E-03	3.6E-04
Chaparral*	-	-	-	-
Douglas-fir*	-	-	-	-
Elm-ash-cottonwood*	-	-	-	-
Fir-spruce	1.6E-03	2.1E-04	9.2E-04	9.7E-05
	3.1E-03	4.0E-04	1.6E-03	4.6E-04
	3.0E-05	3.8E-06	2.0E-05	7.4E-06
	4.9E-03	2.3E-04	6.3E-04	7.8E-04
	3.5E-03	2.9E-04	1.2E-03	3.9E-04
	1.3E-03	1.1E-04	2.7E-04	7.5E-05
	2.6E-03	2.8E-04	8.7E-04	2.5E-04
	2.9E-03	3.2E-04	9.2E-04	2.6E-04
	2.7E-03	2.7E-04	1.0E-03	2.8E-04
Hemlock-Sitka spruce*	-	-	-	-
Larch*	-	-	-	-
Loblolly-shortleaf pine*	-	-	-	-

Table S 2: Whole tree chemistry. Values obtained considering the nutrient content and fraction of different tree compartments (equation SI-01). Each row represents the calculated fraction of different trees using the Tree Chemistry Database[3]. The raw table is given in the excel supplement.

Tree specie	Ca	Mg	K	P
Unit	[-]			
Lodgepole pine	1.1E-03	1.6E-04	1.9E-04	9.3E-05
	4.0E-03	2.9E-04	5.8E-04	3.1E-04
	3.5E-03	3.2E-04	5.7E-04	3.2E-04
	7.8E-04	1.6E-04	3.7E-04	9.4E-05
Longleaf-slash pine	1.1E-03	1.6E-04	1.9E-04	9.3E-05
	4.0E-03	2.9E-04	5.8E-04	3.1E-04
	3.5E-03	3.2E-04	5.7E-04	3.2E-04
	7.8E-04	1.6E-04	3.7E-04	9.4E-05
Maple-beech-birch	2.0E-03	1.8E-04	5.8E-04	1.0E-04
	2.8E-03	2.6E-04	1.1E-03	2.2E-04
	3.1E-03	3.7E-04	1.1E-03	2.3E-04
	2.6E-03	3.8E-04	8.9E-04	3.0E-04
	1.7E-03	1.9E-04	4.0E-04	9.8E-05
	2.2E-03	2.8E-04	6.4E-04	2.7E-04
	2.1E-03	2.3E-04	1.1E-03	2.7E-04
Oak-gum-cypress	2.7E-03	3.2E-04	8.5E-04	2.6E-04
Oak-hickory*	-	-	-	-
Oak-pine	1.1E-03	1.6E-04	1.9E-04	9.3E-05
	4.0E-03	2.9E-04	5.8E-04	3.1E-04
	3.5E-03	3.2E-04	5.7E-04	3.2E-04
	7.8E-04	1.6E-04	3.7E-04	9.4E-05
Pinyon-juniper*	-	-	-	-
Ponderosa pine	1.1E-03	1.6E-04	1.9E-04	9.3E-05
	4.0E-03	2.9E-04	5.8E-04	3.1E-04
	3.5E-03	3.2E-04	5.7E-04	3.2E-04

A. Timberland wood composition and nutrient loss

Table S 2: Whole tree chemistry. Values obtained considering the nutrient content and fraction of different tree compartments (equation SI-01). Each row represents the calculated fraction of different trees using the Tree Chemistry Database[3]. The raw table is given in the excel supplement.

Table S 2: Whole tree chemistry. Values obtained considering the nutrient content and fraction of different tree compartments (equation SI-01). Each row represents the calculated fraction of different trees using the Tree Chemistry Database[3]. The raw table is given in the excel supplement.

Tree specie	Ca	Mg	K	P
Unit	[-]			
	7.8E-04	1.6E-04	3.7E-04	9.4E-05
Redwood*	-	-	-	-
Spruce-fir	1.6E-03	2.1E-04	9.2E-04	9.7E-05
	3.1E-03	4.0E-04	1.6E-03	4.6E-04
	3.0E-05	3.8E-06	2.0E-05	7.4E-06
	4.9E-03	2.3E-04	6.3E-04	7.8E-04
	3.5E-03	2.9E-04	1.2E-03	3.9E-04
	1.3E-03	1.1E-04	2.7E-04	7.5E-05
	2.6E-03	2.8E-04	8.7E-04	2.5E-04
	2.9E-03	3.2E-04	9.2E-04	2.6E-04
	2.7E-03	2.7E-04	1.0E-03	2.8E-04
Western hardwoods	1.1E-03	1.6E-04	1.9E-04	9.3E-05
	4.0E-03	2.9E-04	5.8E-04	3.1E-04
	3.5E-03	3.2E-04	5.7E-04	3.2E-04
	7.8E-04	1.6E-04	3.7E-04	9.4E-05
Western white pine	1.1E-03	1.6E-04	1.9E-04	9.3E-05
	4.0E-03	2.9E-04	5.8E-04	3.1E-04
	3.5E-03	3.2E-04	5.7E-04	3.2E-04
	7.8E-04	1.6E-04	3.7E-04	9.4E-05
White-red-jack pine	1.1E-03	1.6E-04	1.9E-04	9.3E-05
	4.0E-03	2.9E-04	5.8E-04	3.1E-04
	3.5E-03	3.2E-04	5.7E-04	3.2E-04
Alaska Spruce-birch	2.6E-03	3.8E-04	8.9E-04	3.0E-04
	1.7E-03	1.9E-04	4.0E-04	9.8E-05
	2.2E-03	2.8E-04	6.4E-04	2.7E-04

Tree specie	Ca	Mg	K	P
Unit	[-]			
	2.1E-03	2.3E-04	1.1E-03	2.7E-04
	4.9E-03	2.3E-04	6.3E-04	7.8E-04
	3.5E-03	2.9E-04	1.2E-03	3.9E-04
	1.3E-03	1.1E-04	2.7E-04	7.5E-05
	2.6E-03	2.8E-04	8.7E-04	2.5E-04
	2.9E-03	3.2E-04	9.2E-04	2.6E-04
	2.7E-03	2.7E-04	1.0E-03	2.8E-04
All trees	1.6E-03	2.1E-04	9.2E-04	9.7E-05
	3.1E-03	4.0E-04	1.6E-03	4.6E-04
	1.1E-03	1.6E-04	1.9E-04	9.3E-05
	4.0E-03	2.9E-04	5.8E-04	3.1E-04
	3.5E-03	3.2E-04	5.7E-04	3.2E-04
	7.8E-04	1.6E-04	3.7E-04	9.4E-05
	2.0E-03	1.8E-04	5.8E-04	1.0E-04
	2.8E-03	2.6E-04	1.1E-03	2.2E-04
	3.1E-03	3.7E-04	1.1E-03	2.3E-04
	1.7E-03	1.9E-04	4.0E-04	9.8E-05
	2.2E-03	2.8E-04	6.4E-04	2.7E-04
	2.1E-03	2.3E-04	1.1E-03	2.7E-04
	3.0E-05	3.8E-06	2.0E-05	7.4E-06
	4.9E-03	2.3E-04	6.3E-04	7.8E-04
	3.5E-03	2.9E-04	1.2E-03	3.9E-04
	1.3E-03	1.1E-04	2.7E-04	7.5E-05
	2.6E-03	2.8E-04	8.7E-04	2.5E-04
	2.9E-03	3.2E-04	9.2E-04	2.6E-04

A. Timberland wood composition and nutrient loss

Table S 2: Whole tree chemistry. Values obtained considering the nutrient content and fraction of different tree compartments (equation SI-01). Each row represents the calculated fraction of different trees using the Tree Chemistry Database[3]. The raw table is given in the excel supplement.

Tree specie	Ca	Mg	K	P
Unit	[-]			
	2.7E-03	2.7E-04	1.0E-03	2.8E-04
	3.4E-03	4.7E-04	1.8E-03	3.2E-04
	2.7E-03	3.0E-04	8.2E-04	1.4E-04
	5.2E-02	5.8E-04	2.0E-03	3.2E-04
	3.6E-02	8.7E-04	7.1E-03	4.8E-04
	2.0E-02	7.2E-04	1.9E-03	3.5E-04
	3.7E-03	6.7E-04	2.2E-03	3.6E-04
	2.7E-03	3.2E-04	8.5E-04	2.6E-04

*Tree-species without information concerning chemistry or fractions. In these cases, the information concerning all tree-species within interest area, which had values for element fractions and chemistry, was used. All tree-species values are reported in this table as "All trees" and they were used for trees with asterisk (*).

Table S 3: Tree compartment fraction. Values used for quantifying the nutrient fractions for whole-tree harvest. Each row represents a different compartment fraction based from different samples[3]. The raw table is given in the excel supplement.

Tree specie	Dead wood	Stem	Bark	Bole	Branch	Twig	Foliage
Unit	[-]						
Aspen-birch	0.016	0	0.086	0.661	0.215	0	0.022
	0.006	0	0.113	0.646	0.199	0.036	0
	0.021	0	0.107	0.684	0.169	0	0.019
	0	0	0.108	0.727	0.142	0	0.023
	0.051	0	0.152	0.583	0.191	0	0.023
	0.036	0	0.156	0.661	0.124	0.024	0
	0.013	0	0.241	0.607	0.095	0.012	0.032
	0.059	0	0.219	0.487	0.168	0.02	0.047
	0.054	0	0.191	0.591	0.138	0.008	0.018
	0.053	0	0.176	0.584	0.166	0	0.021
Chaparral*	-	-	-	-	-	-	-
Douglas-fir*	-	-	-	-	-	-	-
Elm-ash-cottonwood*	-	-	-	-	-	-	-
Fir-spruce	0.067	0	0.082	0.559	0.12	0.172	0
	0.06	0.018	0.093	0.486	0.188	0	0.158
	0	0	0	0	0.0023	0	0.0022
	0.106	0	0.102	0.402	0.228	0	0.161
	0.12	0	0.05	0.56	0.15	0	0.12
	0.125	0	0.061	0.486	0.162	0.167	0
	0.079	0.014	0.08	0.636	0.114	0	0.077
	0.089	0.015	0.084	0.575	0.158	0	0.085
	0.047	0.018	0.091	0.621	0.128	0	0.095
Hemlock-Sitka spruce*	-	-	-	-	-	-	-

A. Timberland wood composition and nutrient loss

Table S 3: Tree compartment fraction. Values used for quantifying the nutrient fractions for whole-tree harvest. Each row represents a different compartment fraction based from different samples[3]. The raw table is given in the excel supplement.

Tree specie	Dead wood	Stem	Bark	Bole	Branch	Twig	Foliage
Unit				[-]			
Larch*	-	-	-	-	-	-	-
Loblolly-shortleaf pine*	-	-	-	-	-	-	-
Lodgepole pine	0.017	0	0.063	0.756	0.114	0.05	0
	0.06	0	0.1	0.57	0.18	0	0.09
	0.106	0	0.119	0.542	0.137	0	0.096
	0.065	0	0.086	0.609	0.178	0.061	0
Longleaf-slash pine	0.017	0	0.063	0.756	0.114	0.05	0
	0.06	0	0.1	0.57	0.18	0	0.09
	0.106	0	0.119	0.542	0.137	0	0.096
	0.065	0	0.086	0.609	0.178	0.061	0
Maple-beech-birch	0.023	0	0.085	0.64	0.198	0.055	0
	0.032	0	0.088	0.68	0.18	0	0.02
	0.025	0	0.086	0.684	0.185	0	0.02
	0.016	0	0.086	0.661	0.215	0	0.022
	0.006	0	0.113	0.646	0.199	0.036	0
	0.021	0	0.107	0.684	0.169	0	0.019
	0	0	0.108	0.727	0.142	0	0.023
Oak-gum-cypress	0	0.033	0.078	0.801	0.088	0	0
Oak-hickory*	-	-	-	-	-	-	-
Oak-pine	0.017	0	0.063	0.756	0.114	0.05	0
	0.06	0	0.1	0.57	0.18	0	0.09
	0.106	0	0.119	0.542	0.137	0	0.096
	0.065	0	0.086	0.609	0.178	0.061	0

Table S 3: Tree compartment fraction. Values used for quantifying the nutrient fractions for whole-tree harvest. Each row represents a different compartment fraction based from different samples[3]. The raw table is given in the excel supplement.

Tree specie	Dead wood	Stem	Bark	Bole	Branch	Twig	Foliage
Unit				[-]			
Pinyon-juniper*	-	-	-	-	-	-	-
Ponderosa pine	0.017	0	0.063	0.756	0.114	0.05	0
	0.06	0	0.1	0.57	0.18	0	0.09
	0.106	0	0.119	0.542	0.137	0	0.096
	0.065	0	0.086	0.609	0.178	0.061	0
Redwood*	-	-	-	-	-	-	-
Spruce-fir	0.067	0	0.082	0.559	0.12	0.172	0
	0.06	0.018	0.093	0.486	0.188	0	0.158
	0	0	0	0	0.0023	0	0.0022
	0.106	0	0.102	0.402	0.228	0	0.161
	0.12	0	0.05	0.56	0.15	0	0.12
	0.125	0	0.061	0.486	0.162	0.167	0
	0.079	0.014	0.08	0.636	0.114	0	0.077
	0.089	0.015	0.084	0.575	0.158	0	0.085
	0.047	0.018	0.091	0.621	0.128	0	0.095
Western hardwoods	0.017	0	0.063	0.756	0.114	0.05	0
	0.06	0	0.1	0.57	0.18	0	0.09
	0.106	0	0.119	0.542	0.137	0	0.096
	0.065	0	0.086	0.609	0.178	0.061	0
Western white pine	0.017	0	0.063	0.756	0.114	0.05	0
	0.06	0	0.1	0.57	0.18	0	0.09
	0.106	0	0.119	0.542	0.137	0	0.096
	0.065	0	0.086	0.609	0.178	0.061	0
White-red-jack	0.017	0	0.063	0.756	0.114	0.05	0

A. Timberland wood composition and nutrient loss

Table S 3: Tree compartment fraction. Values used for quantifying the nutrient fractions for whole-tree harvest. Each row represents a different compartment fraction based from different samples[3]. The raw table is given in the excel supplement.

Tree specie	Dead wood	Stem	Bark	Bole	Branch	Twig	Foliage
Unit				[-]			
pine	0.06	0	0.1	0.57	0.18	0	0.09
	0.106	0	0.119	0.542	0.137	0	0.096
Alaska Spruce-birch	0.016	0	0.086	0.661	0.215	0	0.022
	0.006	0	0.113	0.646	0.199	0.036	0
	0.021	0	0.107	0.684	0.169	0	0.019
	0	0	0.108	0.727	0.142	0	0.023
	0.106	0	0.102	0.402	0.228	0	0.161
	0.12	0	0.05	0.56	0.15	0	0.12
	0.125	0	0.061	0.486	0.162	0.167	0
	0.079	0.014	0.08	0.636	0.114	0	0.077
	0.089	0.015	0.084	0.575	0.158	0	0.085
	0.047	0.018	0.091	0.621	0.128	0	0.095
All trees	0.067	0	0.082	0.559	0.12	0.172	0
	0.06	0.018	0.093	0.486	0.188	0	0.158
	0.017	0	0.063	0.756	0.114	0.05	0
	0.06	0	0.1	0.57	0.18	0	0.09
	0.106	0	0.119	0.542	0.137	0	0.096
	0.065	0	0.086	0.609	0.178	0.061	0
	0.023	0	0.085	0.64	0.198	0.055	0
	0.032	0	0.088	0.68	0.18	0	0.02
	0.025	0	0.086	0.684	0.185	0	0.02
	0.006	0	0.113	0.646	0.199	0.036	0
	0.021	0	0.107	0.684	0.169	0	0.019

Table S 3: Tree compartment fraction. Values used for quantifying the nutrient fractions for whole-tree harvest. Each row represents a different compartment fraction based from different samples[3]. The raw table is given in the excel supplement.

Tree specie	Dead wood	Stem	Bark	Bole	Branch	Twig	Foliage
Unit				[-]			
	0	0	0.108	0.727	0.142	0	0.023
	0	0	0	0	0.0023	0	0.0022
	0.106	0	0.102	0.402	0.228	0	0.161
	0.12	0	0.05	0.56	0.15	0	0.12
	0.125	0	0.061	0.486	0.162	0.167	0
	0.079	0.014	0.08	0.636	0.114	0	0.077
	0.089	0.015	0.084	0.575	0.158	0	0.085
	0.047	0.018	0.091	0.621	0.128	0	0.095
	0.051	0	0.152	0.583	0.191	0	0.023
	0.036	0	0.156	0.661	0.124	0.024	0
	0.013	0	0.241	0.607	0.095	0.012	0.032
	0.059	0	0.219	0.487	0.168	0.02	0.047
	0.054	0	0.191	0.591	0.138	0.008	0.018
	0.053	0	0.176	0.584	0.166	0	0.021
	0	0.033	0.078	0.801	0.088	0	0

*Tree-species without information concerning chemistry or fractions. In these cases, information concerning all tree-species within interest area, which had values for element fractions and chemistry, was used. All tree-species values are reported in this table as "All trees" and they were used for obtaining the 25th/75th and median nutrient values of trees marked by an asterisk (*).

B. Nutrient Supply

For timberland areas, total atmospheric deposition rates and weathering nutrient supply are considered as different nutrient sources. For regions in which the weathering rates reported by Hartmann et. al.[5] were low (Fig. S 7), or depending on the lithological class geochemistry, atmospheric deposition is the main Ca, Mg, K, and P source to the system. To show the nutrient contribution from the different sources, maps for the difference between the atmospheric deposition rates and weathering nutrient fluxes are presented (Fig. S 9a – Fig. S 16a). Special attention was given to the nutrient source contribution within timberland areas. Extra frequency diagrams presenting the atmospheric or weathering nutrient contribution within timberland areas are given in Fig. S 9b to Fig. S 16b.

B1) Total atmospheric deposition

Total (wet + dry) atmospheric chemical deposition maps from the National Atmospheric Deposition Program[6] (cf. Schwede[7]) were used to quantify the median U.S. continental atmospheric deposition. Ca, Mg, and K were the considered elements for a deposition range for years 2000 to 2015 (Fig. S 3 to Fig. S 5) was considered. The respective Mg, Ca, and K minima and maxima deposition values are: 5.5 – 1.7×10^{3}, 21 – 2.7×10^{3}, and 6.2 – 2.7×10^{2} kg km^{-2} a^{-1}. The atmospheric deposition is spatially heterogeneous, but patterns are identifiable. For Ca, the highest depositions occur, in their majority, within longitudes 100ºW and 120ºW. While the atmospheric deposition for K is higher on the U.S. Eastern part, comprehending longitudes between 80ºW and 120ºW. For Mg, the highest atmospheric deposition concentrations are observed over the entire U.S. coast. Phosphorus total deposition[8] was obtained from a global simulation. The reported depositional values within U.S. were 0.1 – 4.1 kg km^{-2} a^{-1}, with the highest values located on the Eastern U.S. (Fig. S 6).

Resulting median Ca, Mg, K, and P raster files (Fig. S 3 to Fig. S 6) were overlaid to the timberland distribution maps (Fig. S 2) and to the GLIM database to obtain the interest area atmospheric deposition values. By this, it was possible to quantify the spatially-explicit and averaged atmospheric nutrient deposition rates within each lithological class

B. Nutrient Supply

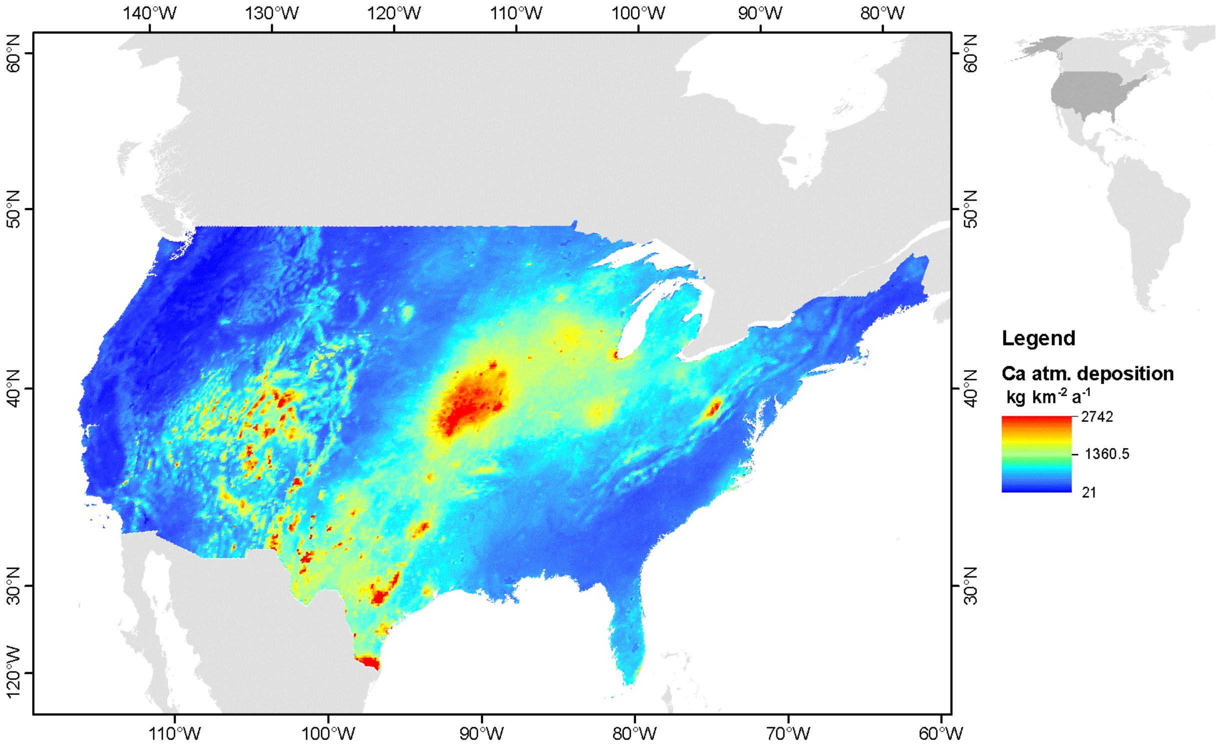

Figure S 3: Total atmospheric Ca deposition [kg km^{-1} a^{-1}] for U.S.[6]. Map generated with ESRI ArcGIS ver. 10.3.1 (http://www.esri.com).

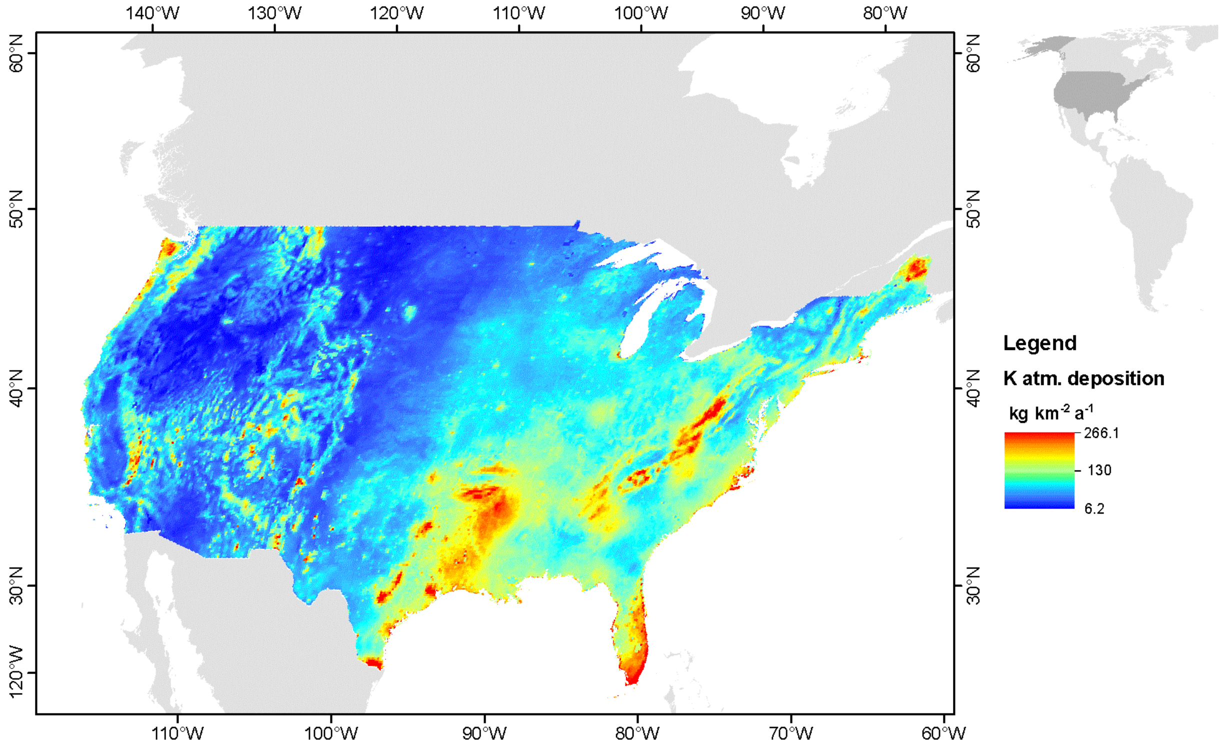

Figure S 4: Total atmospheric K deposition [kg km^{-1} a^{-1}] for U.S.[6]. Map generated with ESRI ArcGIS ver. 10.3.1 (http://www.esri.com).

B. Nutrient Supply

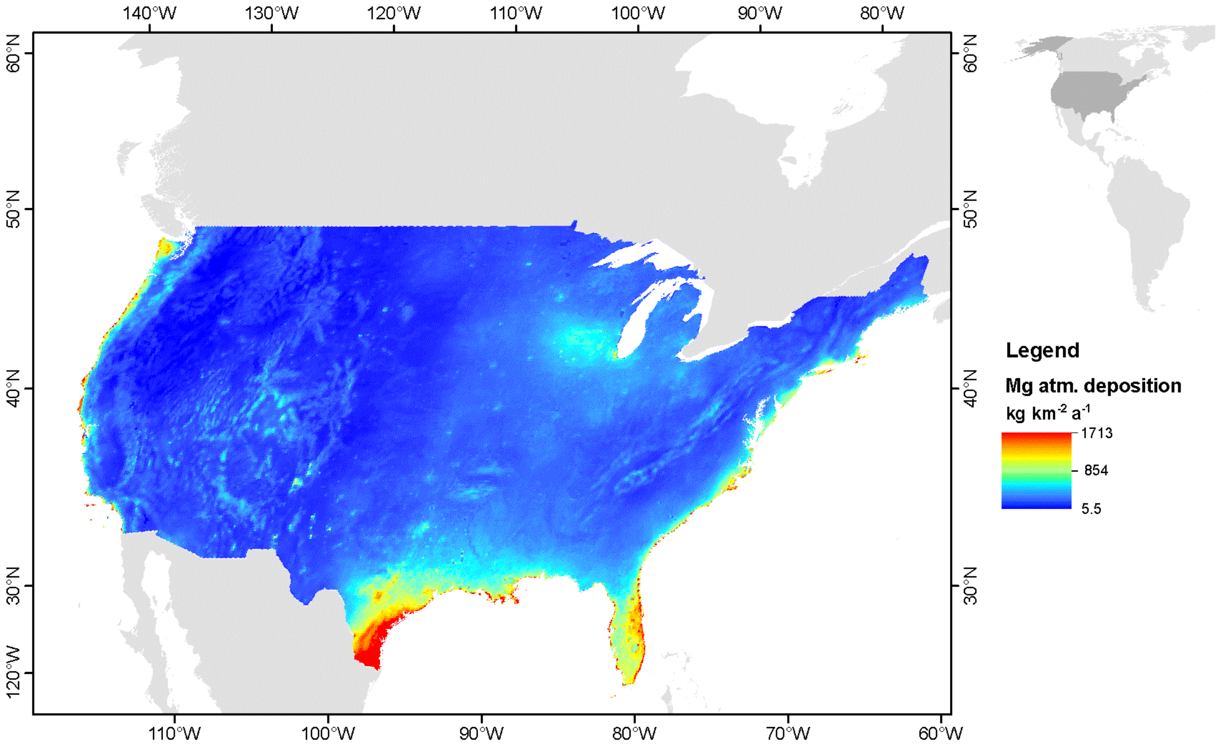

Figure S 5: Total atmospheric Mg deposition [kg km^{-1} a^{-1}] for U.S.[6]. Map generated with ESRI ArcGIS ver. 10.3.1 (http://www.esri.com).

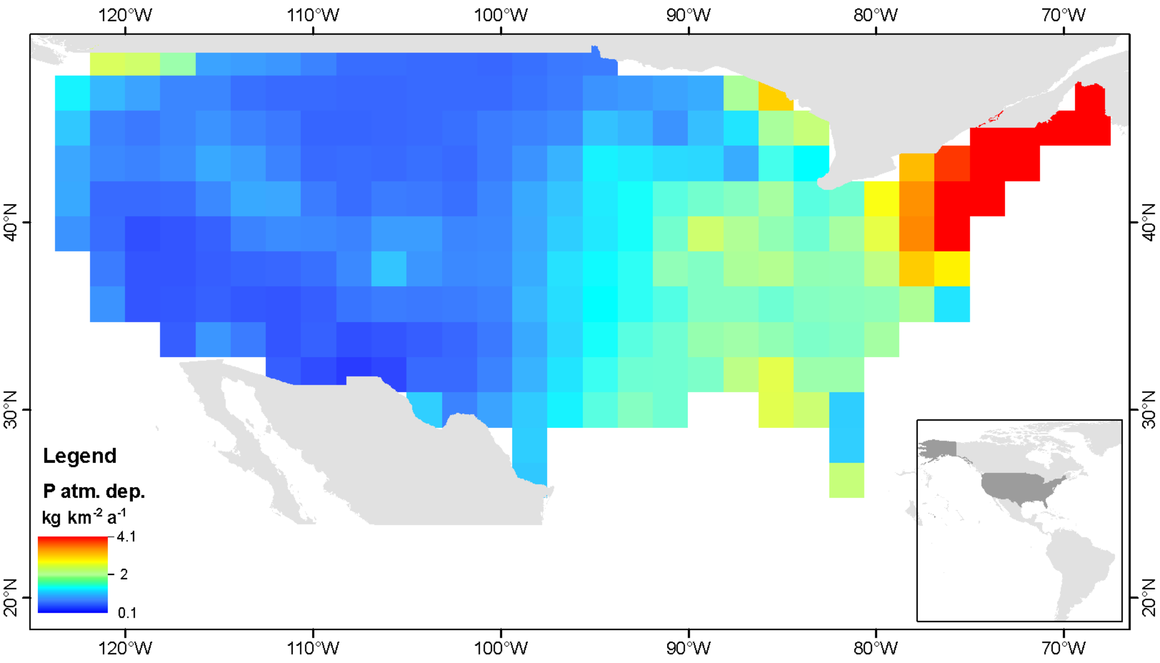

Figure S 6: Atmospheric P deposition [kg km^{-1} a^{-1}] for U.S. extracted from[8]. Map generated with ESRI ArcGIS ver. 10.3.1 (http://www.esri.com).

B. Nutrient Supply

B2) Weathering Rates (WR)

Weathering rates were used (corresponding to equation (3) WR_{calc} in the main text) from the Hartmann et al.[5] model framework. Weathering rates provide a maximum and minimum range, representing fresh and easily weatherable lithology (no soil shielding effect) and locations where weathering is, by soil shielding effects, comparable to humid tropical conditions with depleted soils overlaying the considered lithology. Weathering rates were obtained by overlaying the timberland area shapefile (black areas Fig. S 1) to the weathering rate raster file (Fig. S 7).

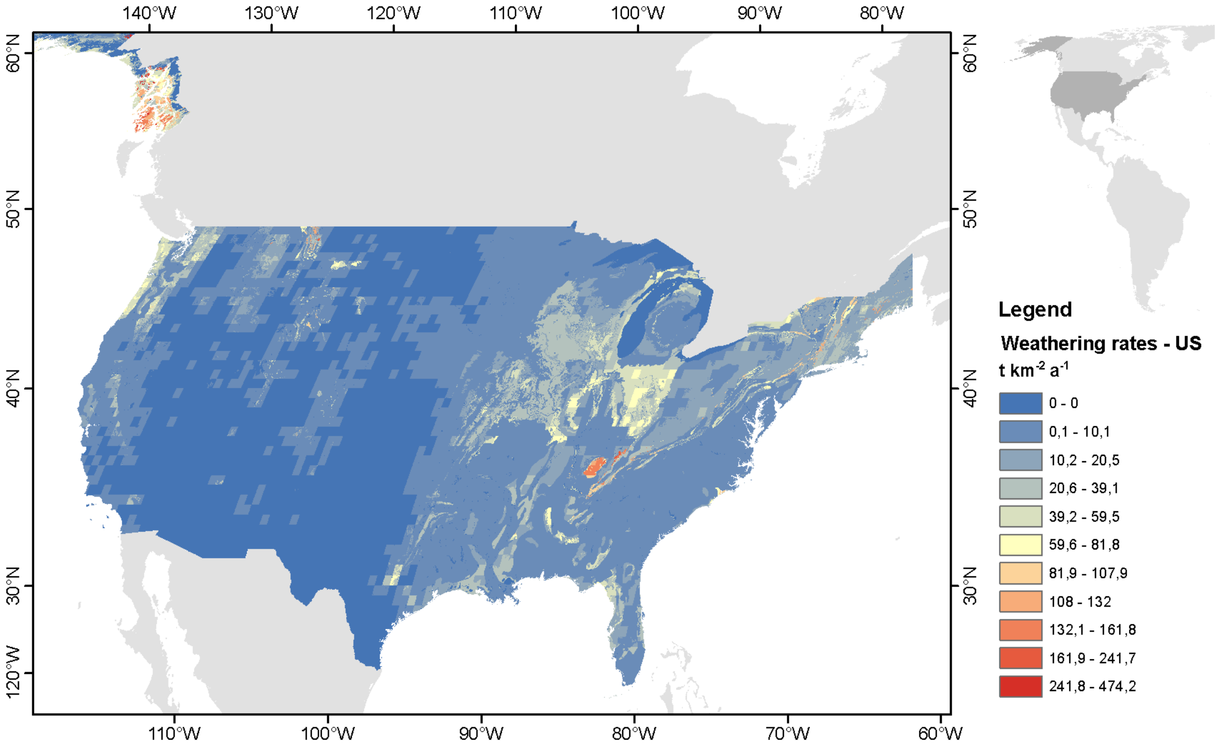

Figure S 7: Weathering rates [t km^{-1} a^{-1}] across continental U.S. obtained from Hartman et al.[5] (WR as Si+Ca+Mg+Na+K). Map generated with ESRI ArcGIS ver. 10.3.1 (http://www.esri.com).

B3) Lithological classes geochemical composition

Spatial rock class distribution within timberland areas for the twelve studied lithological classes was obtained by overlaying the timberland area shapefile (black areas Fig. S 1) to the GLIM database[10]. Typical geochemical composition ranges using median and the 25th/75th percentiles from the GEOROCK database[9] and data for whole-rock analyses from Earthchem webportal (www.earthchem.org) were used. The rock names within GEOROCK database were matched to the rocks within the lithological class description in the Hartmann & Nils[10] work. The used lithological classes were: Unconsolidated sediments (SU), siliciclastic sedimentary rocks (SS), mixed sedimentary rocks (SM), basic volcanic rocks (VB), acid volcanic rocks (VA), basic plutonic rocks (PB), acid plutonic rocks (PA), metamorphic rocks (MT), carbonate sedimentary rocks (SC), pyroclastic rocks (PY), intermediate plutonic rocks (PI) and intermediate volcanic rocks (VI). Results containing the used sample number (n) and concentration ranges are provided in Table S 4. 25th/75th percentiles ranges were used as lower and upper limit for the

discussion, together with the calculated weathering rates $(\mathrm{WR_{calc}})$ from the previous section, for obtaining the nutrient fluxes $\mathrm{Nf_{calc}}$ (Equation 3 in main text).

B4) Nutrient supply by weathering

In this section is presented the steps for calculating the spatially-explicit nutrient supply and for calculating the averaged weathering nutrient supply. Averaged nutrient supply by weathering is used for a generalized discussion as the values consider spatially-explicit nutrient fluxes of timberland areas with low or elevated weathering rates.

Area specific information from the GLIM database[10], timberland area and weathering rates from Hartmann et al.[8] were used to quantify the spatially-explicit weathering nutrient supply (Equation 3 in the main text). The minimal and maximal spatially-explicit nutrient supply by weathering for timberland areas is between 8.5×10^{-3} – 0.2×10^{5}, 4.3×10^{-3} – 1.63×10^{5}, 5.7×10^{-3} – 0.2×10^{5}, and 0.3×10^{-3} – 1.2×10^{3} kg km^{-2} a^{-1} for Mg, Ca, K, and P respectively.

To allow a general discussion (main text Fig. 1), the spatially-explicit values were grouped following the twelve lithological classes. The grouped values considered regions with higher and lower weathering rates for 25th and 75th quartiles of geochemical composition. For each of the twelve lithological classes, the lower weathering nutrient supply boundary was represented by the 25th quartiles of nutrient fluxes. The same was done for obtaining the weathering nutrient supply upper boundary, which considered the 75th quartiles. By this approach, it was possible to predict the nutrient fluxes from areas placed in regions with higher or lower weathering rates.

B5) Geogenic nutrient supply

Geogenic nutrient supply account for the atmospheric and weathering nutrient fluxes together. It was given by the respective sum considering spatially-explicit: a) median atmospheric nutrient deposition values (section B1) to the b) nutrient supply by weathering (section B4), both in kg km^{-2} a^{-1}.

To identify the U.S. areas in which atmospheric or weathering nutrient contribution was higher, maps in which the weathering supply was subtracted from the atmospheric precipitation rates are presented for continental U.S. (Fig. S 9a to Fig. S 16a). Generally, atmospheric contribution is an important source of nutrients for continental U.S.

Looking within timberland areas, nutrient supply by weathering can be more important for forest nutrition than atmospheric nutrient deposition rates (Fig. S 9b to Fig. S 16b). The predominance of weathering supply over the atmospheric precipitation depends on weathering rates and chemical lithology composition. For minima (25th quartile) lithology chemical compositions (Table S 4) atmospheric precipitation is an important timberland nutrient source (Fig. S 10b, Fig. S 12b, Fig. S 14b and Fig. S 16b). If the lithology chemistry changes to the maxima (75th quartile) (Table S 4), nutrient supply by weathering is the main nutrient source to timberlands (Fig. S 9b, Fig. S 11b, Fig. S 13b and Fig. S 15b).

For the diagram presented in the main text Fig. 2, the same steps described in section B4 second paragraph were applied. The difference is that spatially-explicit geogenic information

was used for obtaining the 25th and 75th quartiles, respectively representing upper and lower boundaries. The twelve lithological class's geogenic nutrient supply 25th/75th quartiles and median were compared to measured 25th/75th quartiles and median nutrient fluxes (SI-2) within 115 U.S. catchments[11]. Only catchments composed by at least 95% forest covered area were considered. Due to the monitored catchment's lithological diversity, attributing simple way fluxes to certain lithological classes was not possible. Observed overestimation is attributed to the assumption of complete fresh lithology dissolution assumption and no sink (reactions with and within soil porosity, and erosion). Overestimation occurs for all the lithology classes for geogenic nutrient fluxes when compared to the analyzed watersheds (Fig. S 8).

$$Nf_{catch} = perct(\frac{Q_{riv} * C_e}{A_{catch}}) \quad \text{(SI-2)}$$

Where Nf_{catch} represents the measured nutrient flux [kg km^{-2} a^{-1}], C_e is the measured aqueous concentration of interest element 'e' (Mg, Ca, K or P) [kg km^{-3}], Q_{riv} is the measured river discharge [km^3 a^{-1}] and A_{catch} is the catchment area [km^2]. $perct$ is the calculated 25th/75th percentiles and median.

Table S 4: Chemical composition considering 25th, 75th quartiles and median for each lithological class and the sample number used to calculate the statistics (n values). Abbreviations: cf. this supplement section B3.

Lithological Class	Ca[9]				K[9]				Mg[9]				P[9]			
	[WT%]															
	25th	75th	Med	n	25th	75th	Med	n	25th	75th	Med	n	25th	75th	Med	N
SU	0.1	0.4	0.2	34	0.4	2.4	1.3	36	0.2	0.7	0.3	37	0.02	0.03	0.02	28
SS	0.3	4.5	1.1	7509	0.8	2.5	1.6	7672	0.4	1.5	0.9	7389	0.02	0.08	0.04	7191
SM	0.6	18.9	4.1	8571	0.5	1.9	1.1	8280	0.4	2.4	1.0	8474	0.02	0.06	0.03	7737
SC	0.6	23.7	7.8	5730	0.3	2.1	0.9	5412	0.5	2.7	1.0	5654	0.02	0.07	0.03	5013
VB	2.4	6.6	5.1	1973	0.8	3.1	1.6	2029	0.8	4.2	2.6	1953	0.04	0.12	0.08	1669
VI	2.7	4.8	3.8	1543	1.2	2.8	2.0	1563	0.8	2.2	1.5	1537	0.04	0.08	0.06	1334
VA	1.0	4.2	2.8	1177	1.6	3.7	2.6	1191	0.2	1.8	0.9	1157	0.02	0.07	0.04	932
PB	0.7	2.6	1.3	559	2.6	4.9	4.3	632	0.2	1.0	0.4	542	0.02	0.07	0.03	414
PI	0.7	5.0	2.1	98	0.9	2.5	1.6	245	0.5	3.0	1.1	114	0.02	0.19	0.03	22
PA	0.7	3.6	2.1	100	1.1	2.6	1.7	355	0.5	1.7	1.0	118	0.03	0.05	0.03	19
MT	0.2	7.8	1.7	739	0.2	2.4	1.2	901	0.4	7.5	2.0	696	0.01	0.04	0.02	487
PY	5.4	7.0	6.3	1629	0.6	1.7	1.0	1641	2.5	4.7	3.9	1629	0.05	0.16	0.10	1485

B. Nutrient Supply

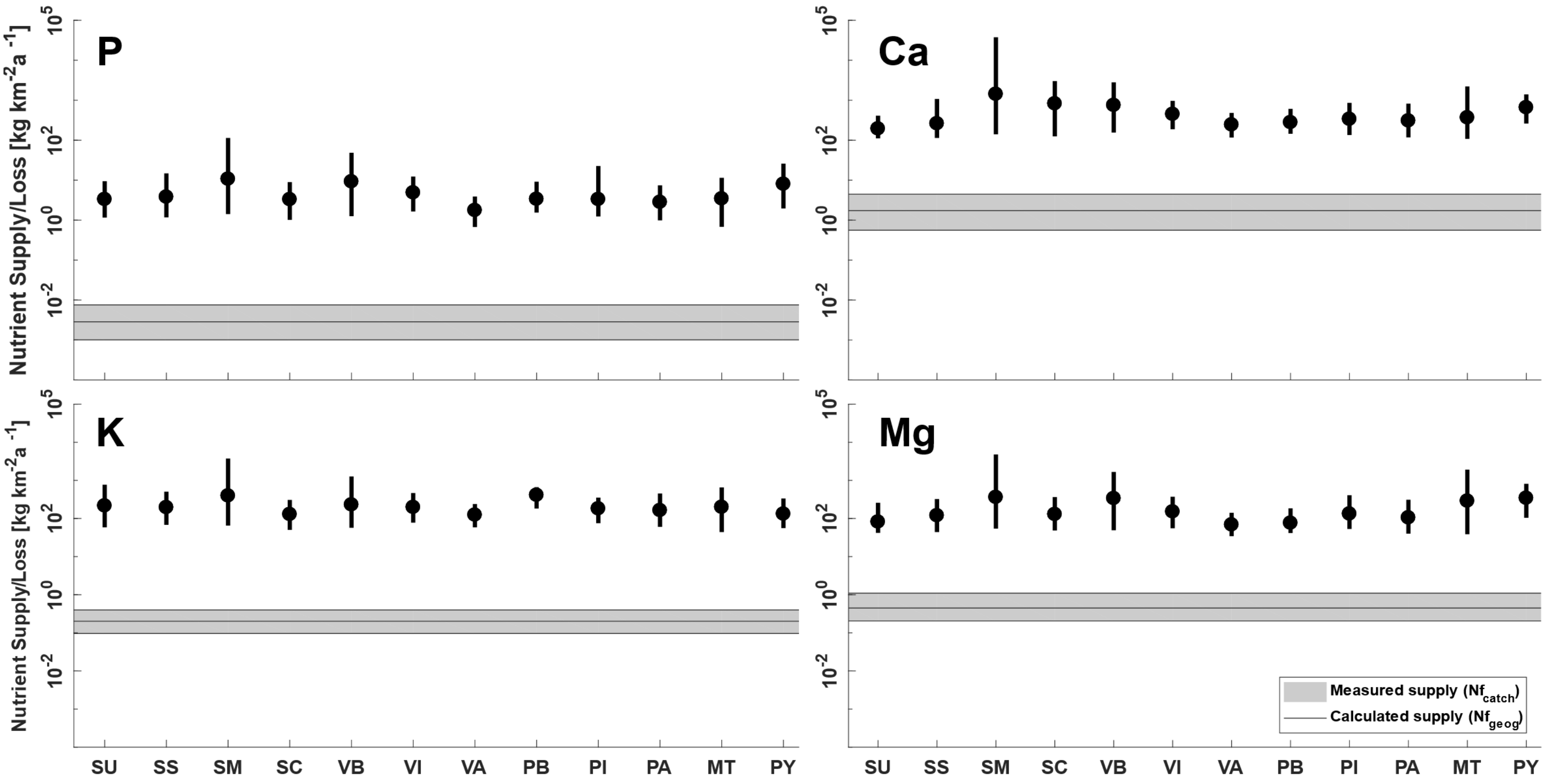

Figure S 8: Comparison between measured and calculated nutrient fluxes. Horizontal lines represent a*veraged measured stream weathering rates* (Nf_{catch}) *for 115 U.S. catchments assuming that river fluxes represent on average long-term losses. Catchments consist of at least 95% of forest covered area*[11]*. Horizontal lines represent minimum median and maximum values. Vertical lines represent total weathering and atmospheric deposition supply* (Nf_{geog}) *following section's B5 steps. Note that the monitored catchment's lithological diversity did not allow for attributing fluxes to certain lithological classes in a simple way.*

Difference between atmospheric deposition and weathering supply

Spatial dependency between atmospheric deposition and weathering supply was investigated. Subtraction of weathering supply from the atmospheric deposition for each considered element was done. For the analysis, two distinct scenarios were investigated considering the median atmospheric deposition and 1) maximum scenario for weathering supply, or 2) minimum scenario for weathering supply (Fig. S 9a to Fig. S 16a). Within timberland areas, the observed pattern occurring for continental U.S. is different. To elucidate this, frequency diagrams considering atmospheric and weathering nutrient supply within timberland areas are presented on the right side of the U.S. maps (Fig. S 9b to Fig. S 16b).

For the timberland areas, considering a maximum weathering supply scenario, the nutrient supply by weathering occurs for 88, 63, 69, and 73% of timberlands for Ca, K, Mg, and P respectively. Considering the minimum scenario for weathering supply, the weathering supply for timberland respectively decreases to 27, 36, 55, and 72% of timberlands for Ca, K, Mg, and P. The nutrient supply by weathering will be the main nutrient source within timberlands depending on factors like lithological element concentration and soil moisture.

Difference between atmospheric deposition and weathering supply

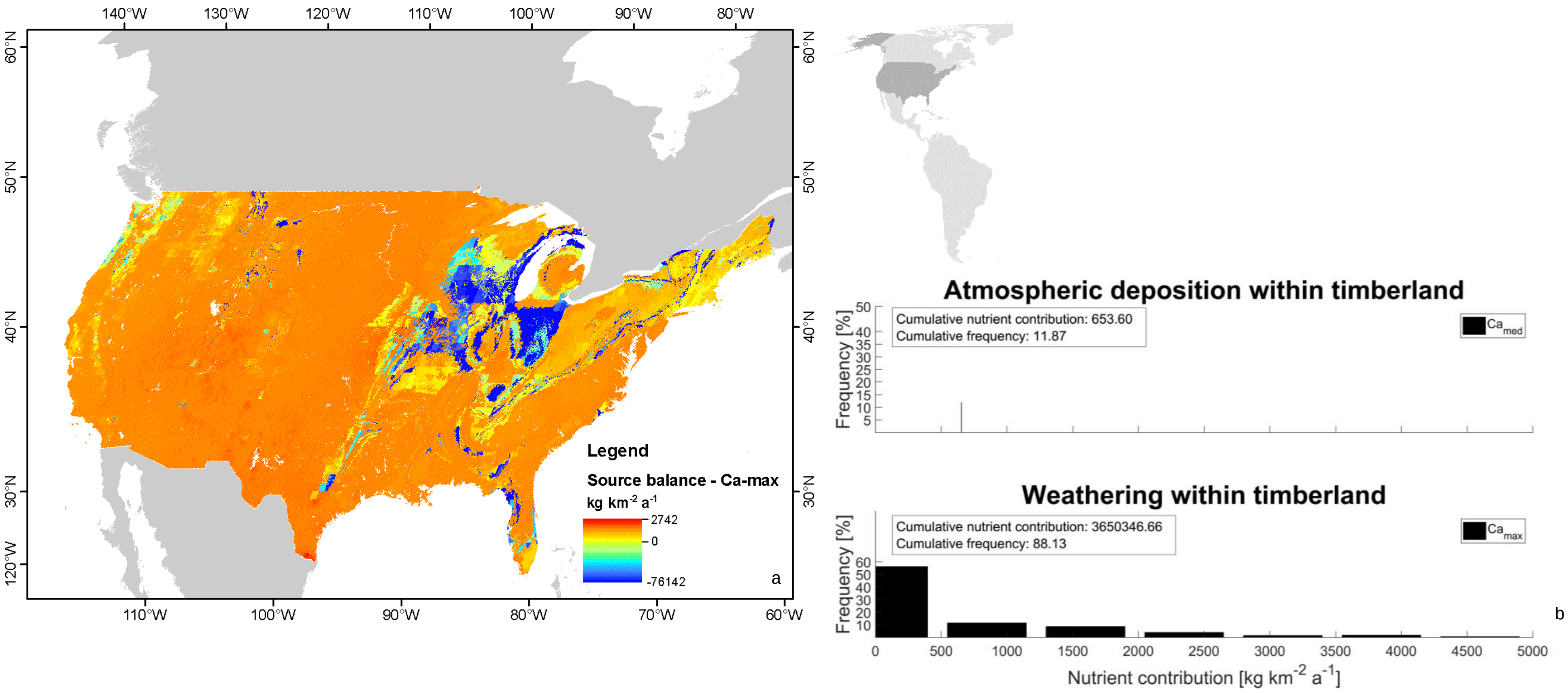

Figure S 9: a) Map representing the result from median Ca atmospheric deposition minus the maximum Ca supply via weathering. Colder colors represent a higher nutrient supply by weathering than atmospheric deposition. Map generated with ESRI ArcGIS ver. 10.3.1 (http://www.esri.com). b) Frequency histograms for the Atmospheric and Weathering nutrient contribution influence within timberland areas considering U.S. difference map. For the weathering nutrient contribution, the values were multiplied by -1 to obtain positive values. Cumulative frequency shows the most contributing nutrient source to timberland nutrition. Weathering and atmospheric cumulative frequency sum up to 100%.

Difference between atmospheric deposition and weathering supply

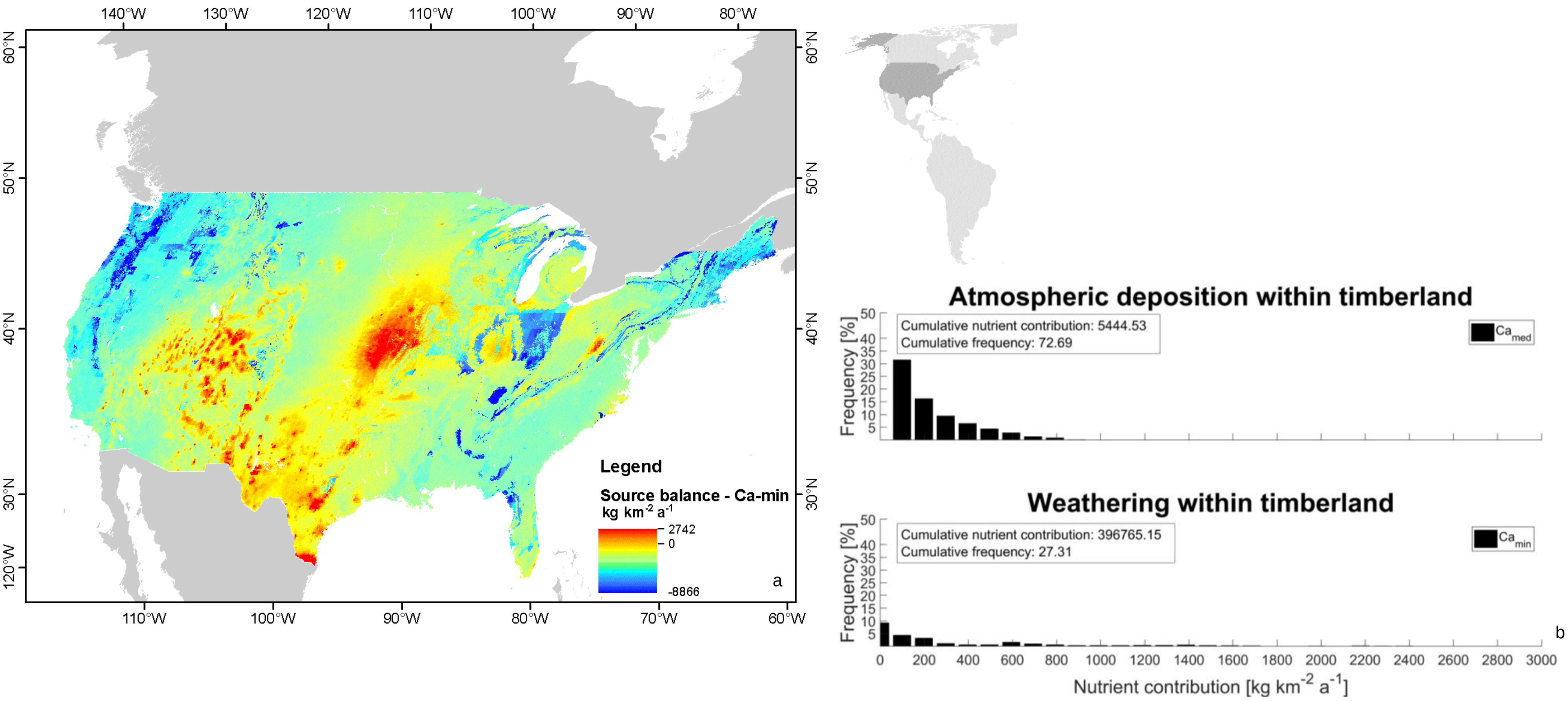

Figure S 10: a) Map representing the result from median Ca atmospheric deposition minus the minimum Ca supply by weathering. Colder colors represent a higher nutrient supply by weathering than atmospheric deposition. Map generated with ESRI ArcGIS ver. 10.3.1 (http://www.esri.com). b) Frequency histograms for the Atmospheric and Weathering nutrient contribution influence within timberland areas considering U.S. difference map. For the weathering nutrient contribution the values were multiplied by -1 to obtain positive values. Cumulative frequency shows the most contributing nutrient source to timberland nutrition. Weathering and atmospheric cumulative frequency sum up to 100%.

Difference between atmospheric deposition and weathering supply

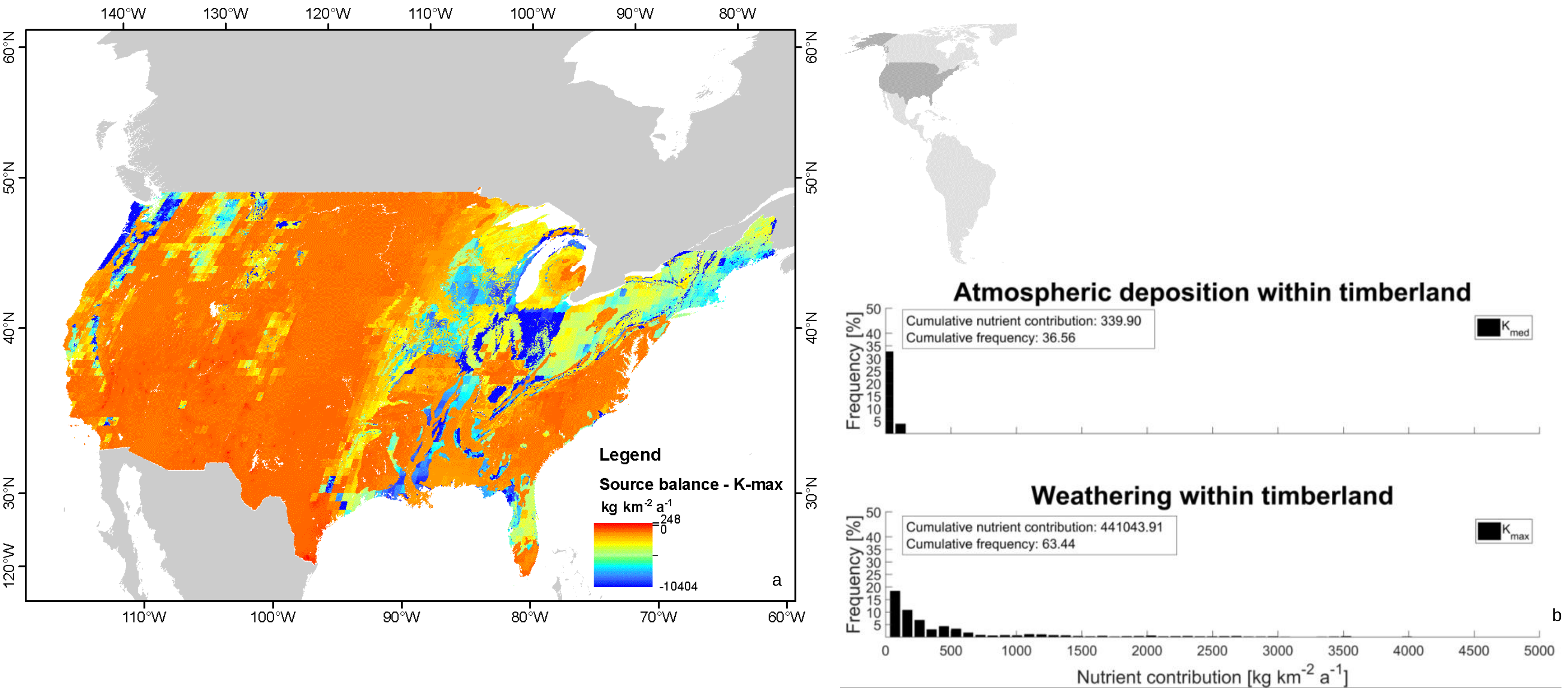

Figure S 11: a) Map representing the result from median K atmospheric deposition minus the maximum K supply by weathering. Colder colors represent a higher nutrient supply by weathering than atmospheric deposition. Map generated with ESRI ArcGIS ver. 10.3.1 (http://www.esri.com). b) Frequency histograms for the Atmospheric and Weathering nutrient contribution influence within timberland areas considering U.S. difference map. For the weathering nutrient contribution the values were multiplied by -1 to obtain positive values. Cumulative frequency shows the most contributing nutrient source to timberland nutrition. Weathering and atmospheric cumulative frequency sum up to 100%.

Difference between atmospheric deposition and weathering supply

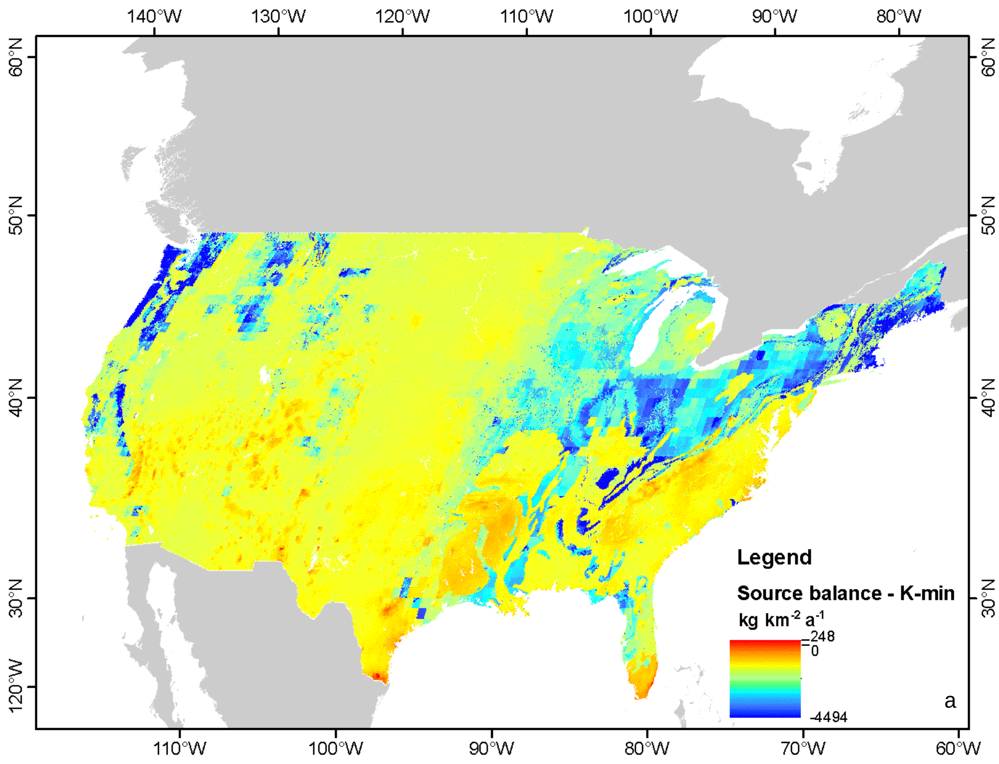

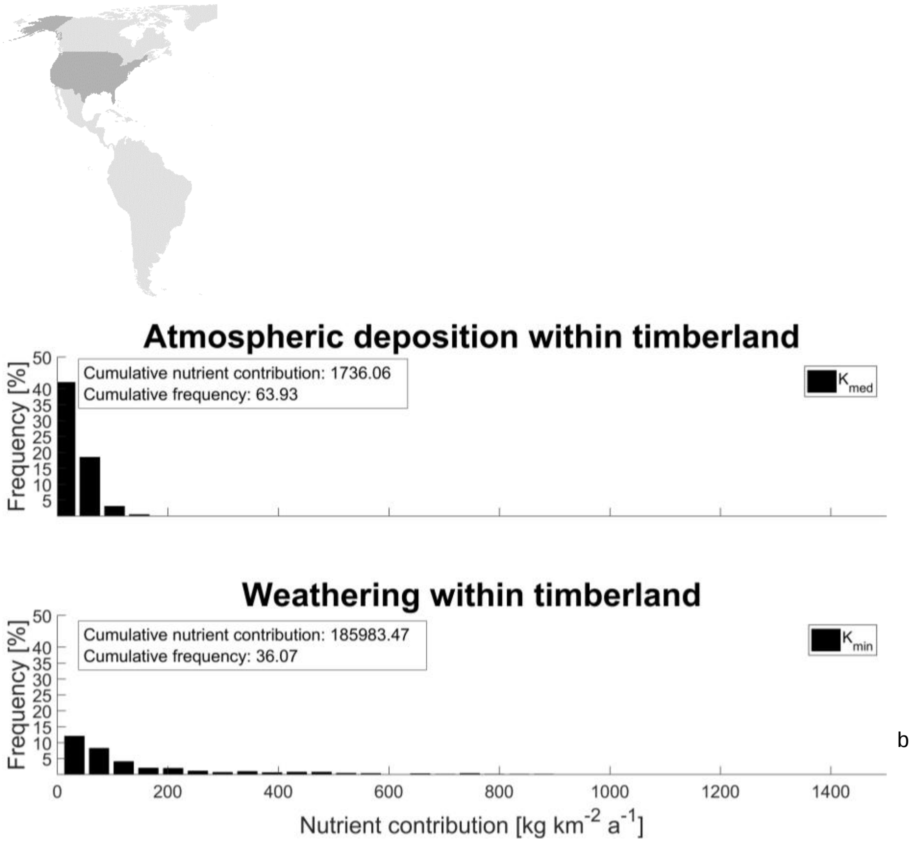

Figure S 12: a) Map representing the result from median K atmospheric deposition minus the minimum K supply by weathering. Colder colors represent a higher nutrient supply by weathering than atmospheric deposition. Map generated with ESRI ArcGIS ver. 10.3.1 (http://www.esri.com). b) Frequency histograms for the Atmospheric and Weathering nutrient contribution influence within timberland areas considering U.S. difference map. For the weathering nutrient contribution the values were multiplied by -1 to obtain positive values. Cumulative frequency shows the most contributing nutrient source to timberland nutrition. Weathering and atmospheric cumulative frequency sum up to 100%.

Difference between atmospheric deposition and weathering supply

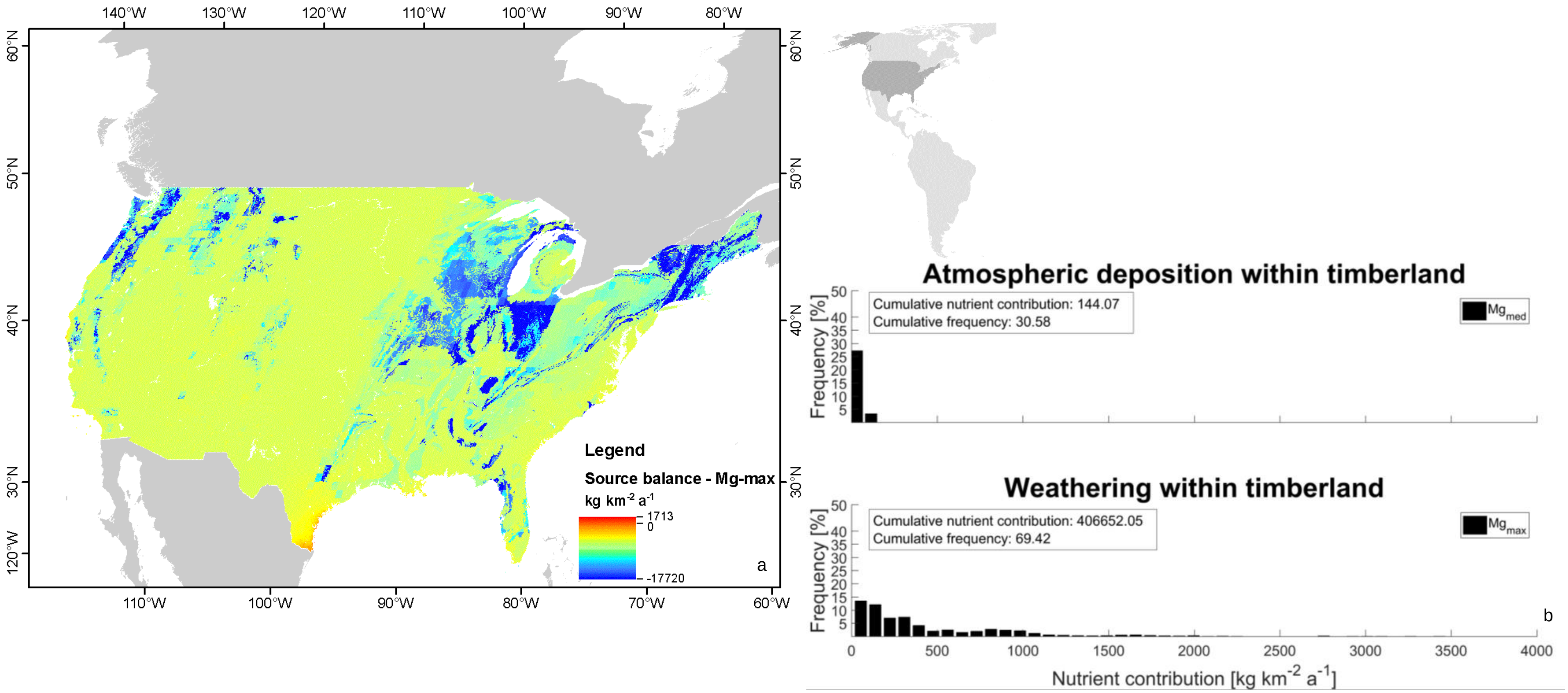

Figure S 13: a) Map representing the result from median Mg atmospheric deposition minus the maximum Mg supply by weathering. Colder colors represent a higher nutrient supply by weathering than atmospheric deposition. Map generated with ESRI ArcGIS ver. 10.3.1 (http://www.esri.com). b) Frequency histograms for the Atmospheric and Weathering nutrient contribution influence within timberland areas considering U.S. difference map. For the weathering nutrient contribution the values were multiplied by -1 to obtain positive values. Cumulative frequency shows the most contributing nutrient source to timberland nutrition. Weathering and atmospheric cumulative frequency sum up to 100%.

Difference between atmospheric deposition and weathering supply

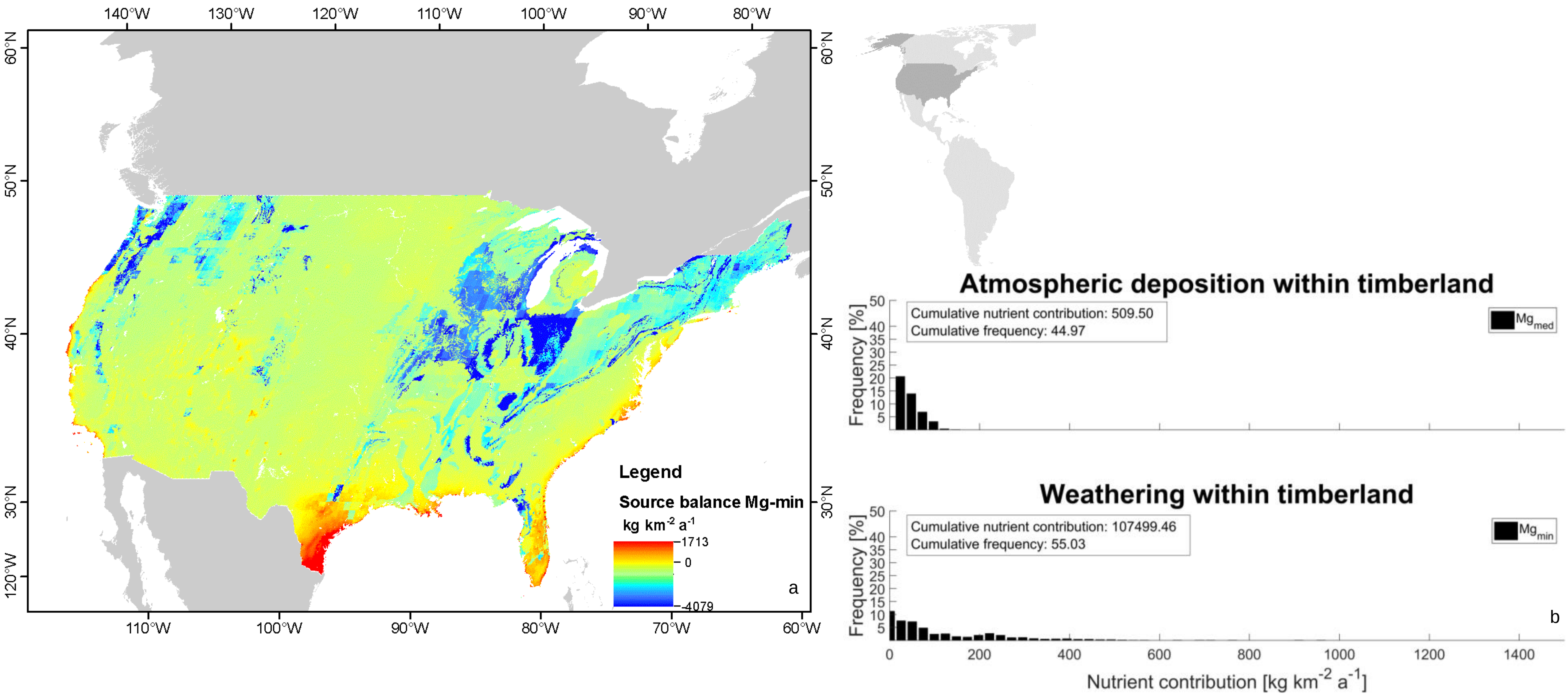

Figure S 14: a) Map representing the result from median Mg atmospheric deposition minus the minimum Mg supply by weathering. Colder colors represent a higher nutrient supply by weathering than atmospheric deposition. Map generated with ESRI ArcGIS ver. 10.3.1 (http://www.esri.com). b) Frequency histograms for the Atmospheric and Weathering nutrient contribution influence within timberland areas considering U.S. difference map. For the weathering nutrient contribution the values were multiplied by -1 to obtain positive values. Cumulative frequency shows the most contributing nutrient source to timberland nutrition. Weathering and atmospheric cumulative frequency sum up to 100%.

Difference between atmospheric deposition and weathering supply

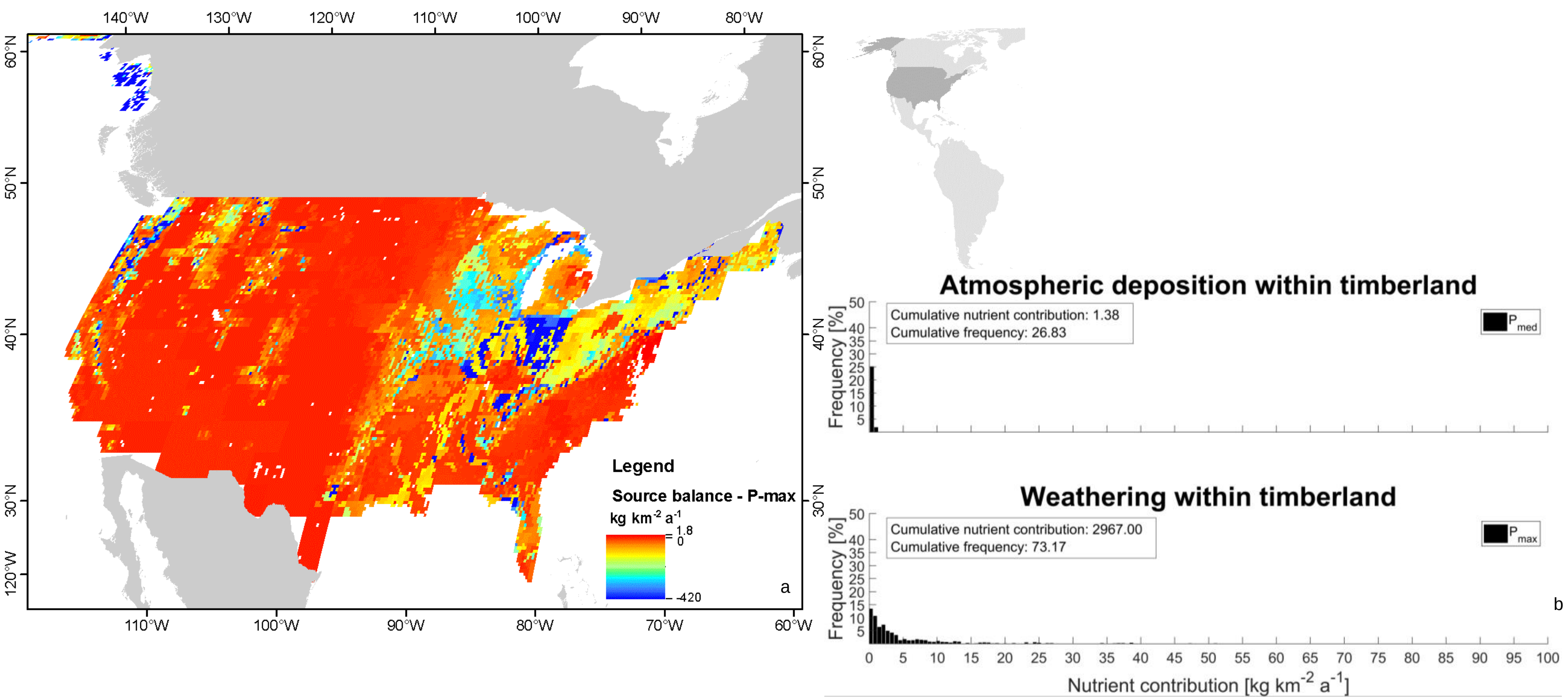

Figure S 15: a) Map representing the result from P atmospheric deposition minus the maximum P supply by weathering. Colder colors represent a higher nutrient supply by weathering than atmospheric deposition. Map generated with ESRI ArcGIS ver. 10.3.1 (http://www.esri.com). b) Frequency histograms for the Atmospheric and Weathering nutrient contribution influence within timberland areas considering U.S. difference map. For the weathering nutrient contribution the values were multiplied by -1 to obtain positive values. Cumulative frequency shows the most contributing nutrient source to timberland nutrition. Weathering and atmospheric cumulative frequency sum up to 100%.

Difference between atmospheric deposition and weathering supply

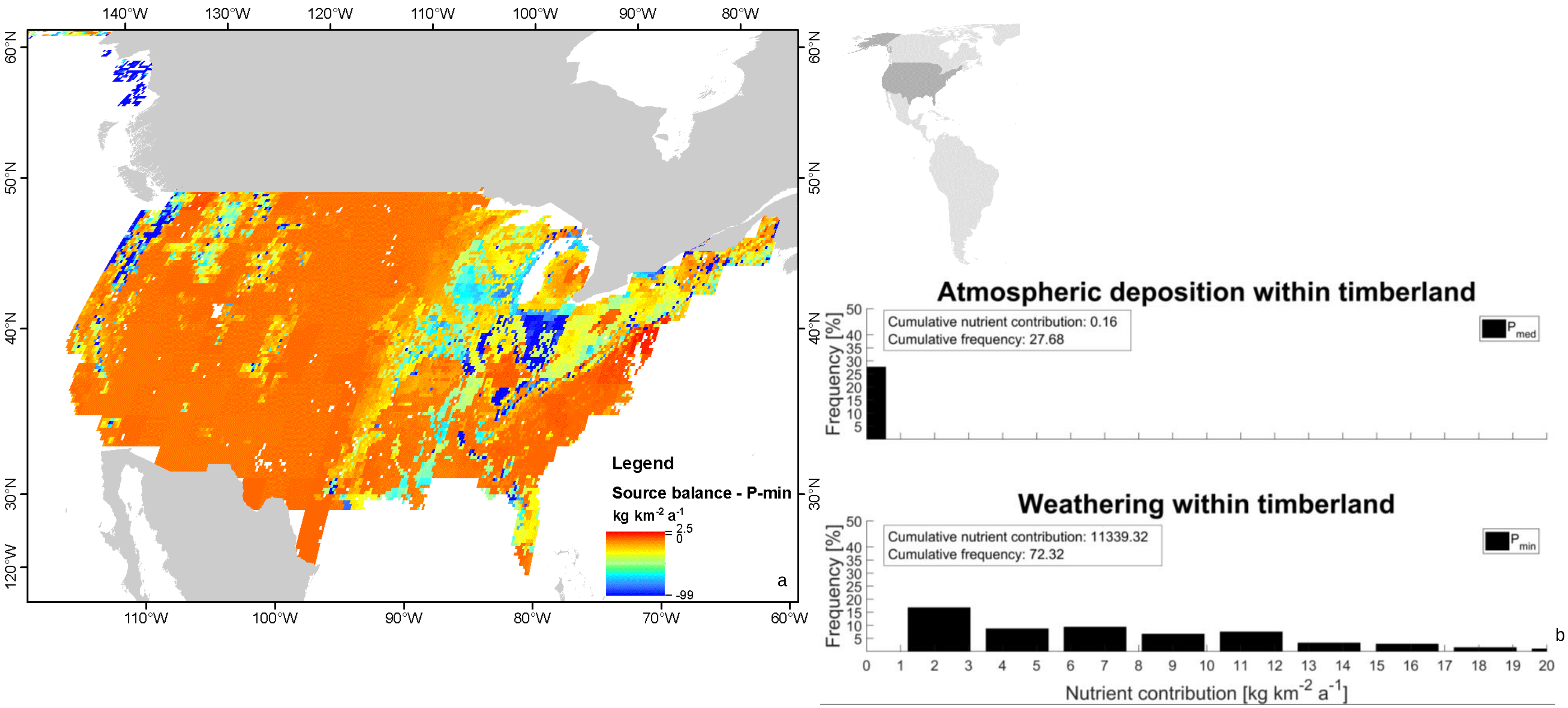

Figure S 16: a) Map representing the result from P atmospheric deposition minus the minimum P supply by weathering. Colder colors represent a higher nutrient supply by weathering than atmospheric deposition. Map generated with ESRI ArcGIS ver. 10.3.1 (http://www.esri.com). b) Frequency histograms for the Atmospheric and Weathering nutrient contribution influence within timberland areas considering U.S. difference map. For the weathering nutrient contribution the values were multiplied by -1 to obtain positive values. Cumulative frequency shows the most contributing nutrient source to timberland nutrition. Weathering and atmospheric cumulative frequency sum up to 100%.

C. Nutrient budget

To assess the nutrient budget, the difference between geogenic nutrient input (weathering nutrient supply plus atmospheric deposition) and nutrient harvest loss was calculated. The nutrient budgets for minimum and maximum scenarios represent the spatially-explicit nutrient losses and supply. The spatially-explicit nutrient budget for Ca, P, Mg, and K (Fig. S 18 to Fig. S 25) reveals the actual nutrient gap for timberland areas considering the spatially-explicit nutrient export based on harvest rates, tree-species and wood density and spatially-explicit total nutrient supply.

To investigate lithological class efficiency in supply nutrients, diagrams considering 25th/75th quartiles and median values for nutrient losses and supply are used. For the spatially averaged diagrams, special attention was given to the lower and upper boundaries of possible nutrient losses and supply. Lower and upper boundaries respectively correspond to scenarios 1 and 8 from Fig. S 17. For this calculation, quantified nutrient losses, as described on section A3, were used, being compared to the nutrient supply by weathering only (section B4 and main text Fig. 1) and geogenic nutrient supply (section B5 and main text Fig. 2).

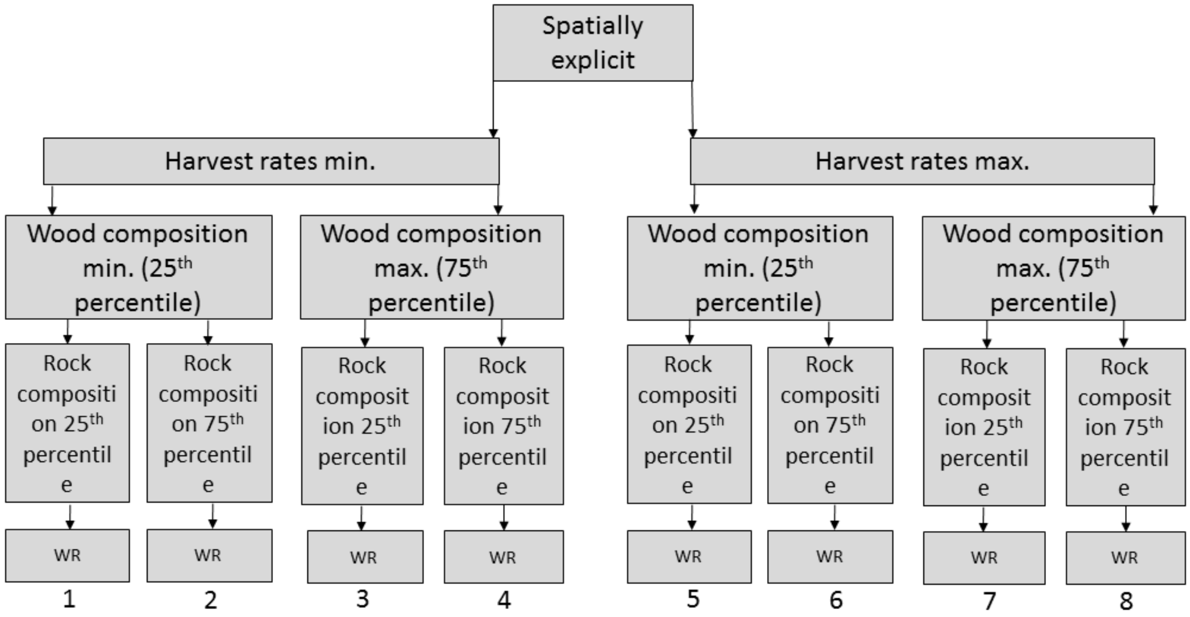

Figure S 17: Nutrient budget scenarios and the respective scenario number. As threshold minimum and maximum, the overall 25th and 75th percentiles for nutrient supply rates based on rock content and for biomass content were chosen at fixed weathering and harvest rates corresponding to scenario 1 and 8 respectively. For $WR = WR_{calc}$ *from*[5].

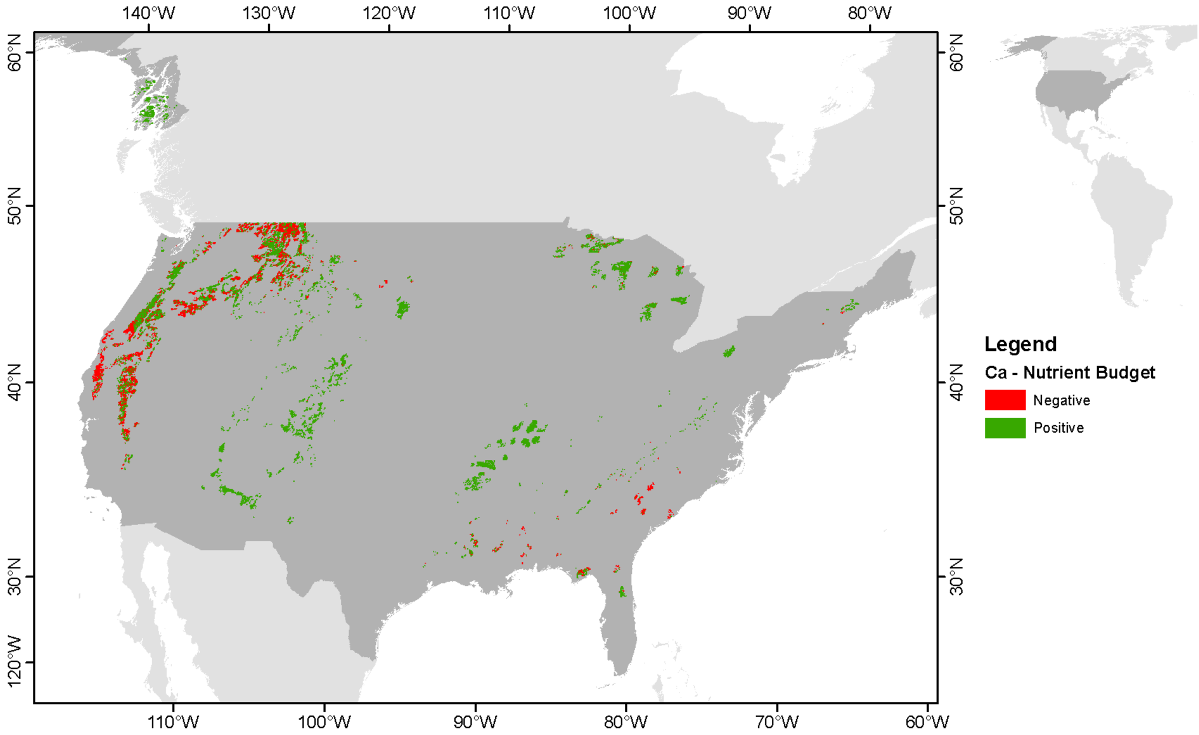

Figure S 18: U.S. timberland spatially-explicit nutrient budget for median atmospheric Ca deposition, and minimum (25th percentile) weathering nutrient supply and harvest loss. Red colors indicate areas with higher harvest nutrient loss than nutrient supply. Green colors represent the opposite. For the shown case, Ca nutrient deficiency occurs for 17% of timberland areas. Map generated with ESRI ArcGIS ver. 10.3.1 (http://www.esri.com).

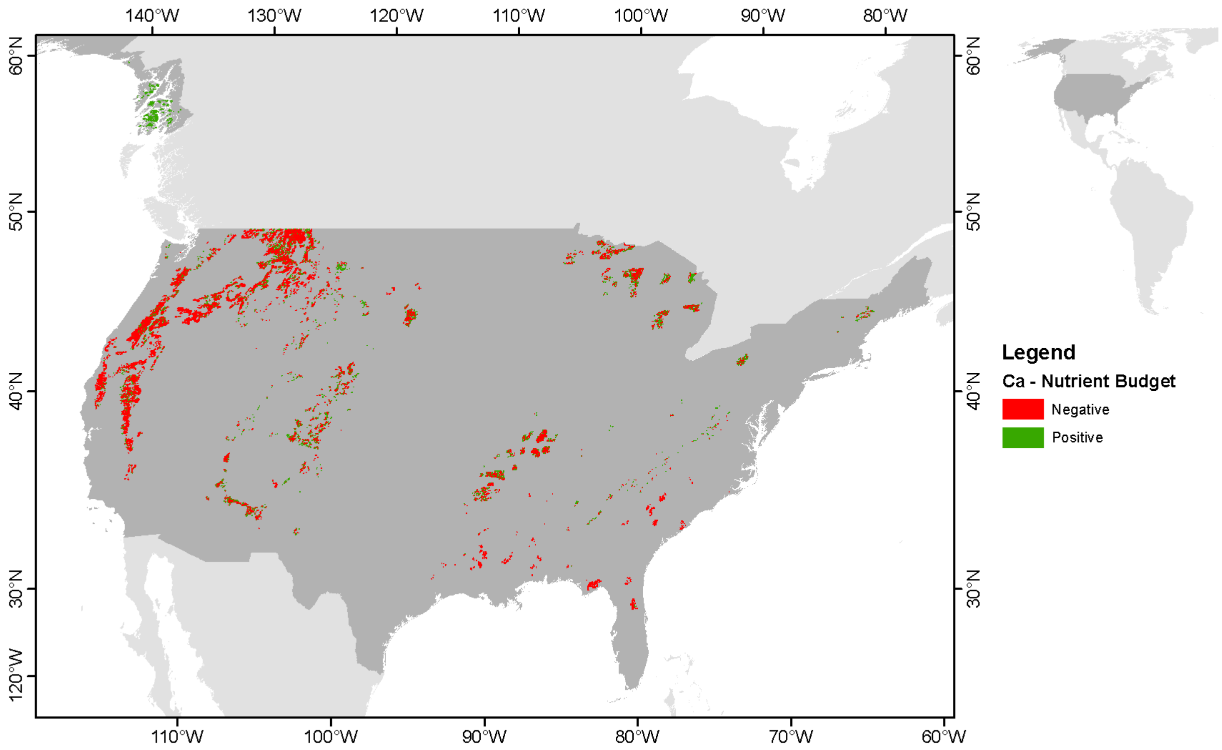

Figure S 19: U.S. timberland spatially-explicit nutrient budget for median atmospheric Ca deposition, and maximum (75th percentile) weathering nutrient supply and harvest loss. Red colors indicate areas with higher harvest nutrient loss than nutrient supply. Green colors represent the opposite. For shown case, Ca nutrient deficiency occurs for 50% of timberland areas. Map generated with ESRI ArcGIS ver. 10.3.1 (http://www.esri.com).

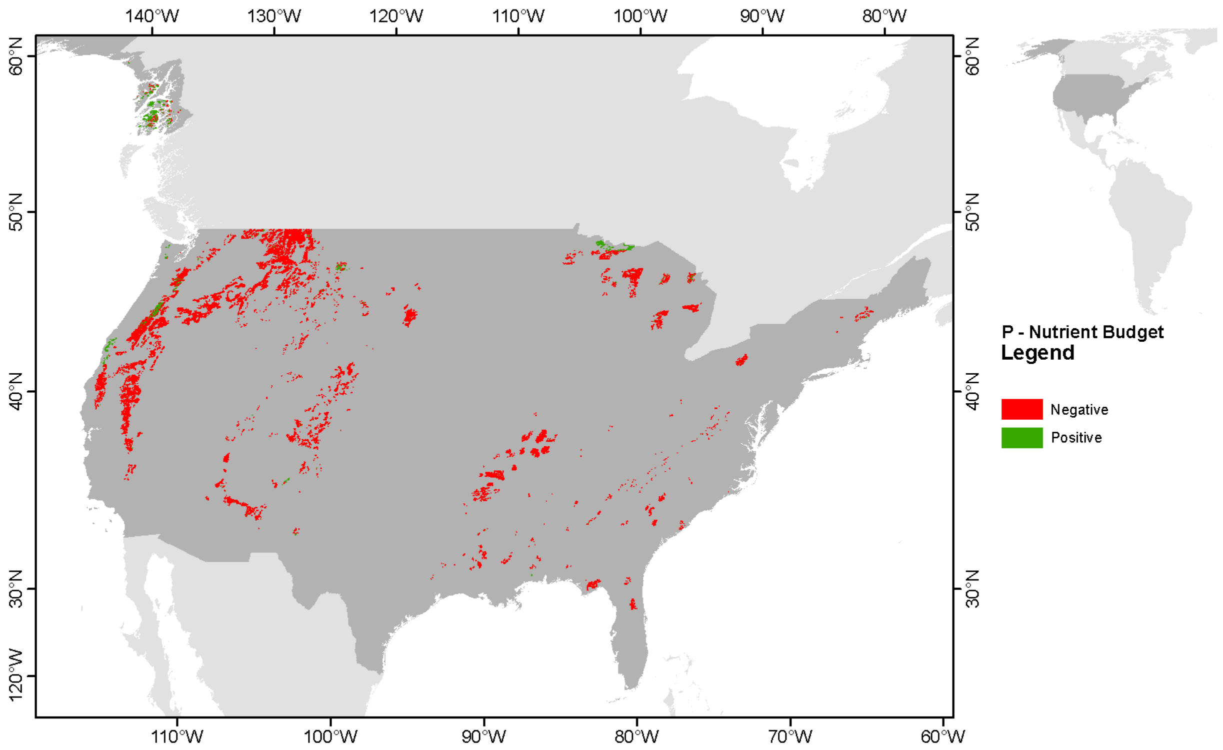

Figure S 20: U.S. timberland spatially-explicit nutrient budget for median atmospheric P deposition, and maximum (75th percentile) geogenic supply and harvest loss. Red colors indicate areas with higher harvest nutrient loss than nutrient supply. Green colors represent the opposite. For shown case, P nutrient deficiency occurs for 96% of timberland areas. Map generated with ESRI ArcGIS ver. 10.3.1 (http://www.esri.com).

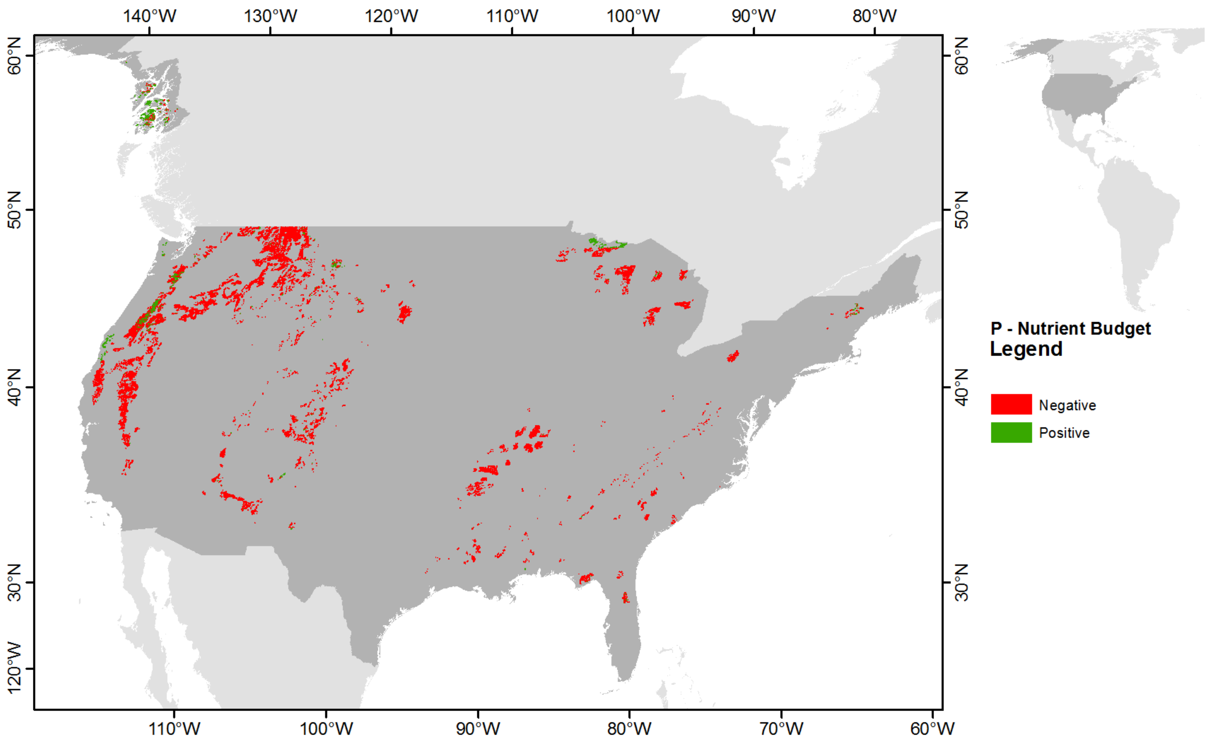

Figure S 21: U.S. timberland spatially-explicit nutrient budget for median atmospheric P deposition, and minimum (25th percentile) geogenic supply and harvest loss. Red colors indicate areas with higher harvest nutrient loss than nutrient supply. Green colors represent the opposite. For shown case, P nutrient deficiency occurs for 94% of timberland areas. Map generated with ESRI ArcGIS ver. 10.3.1 (http://www.esri.com).

Nutrient Budget for elements using two different scenarios

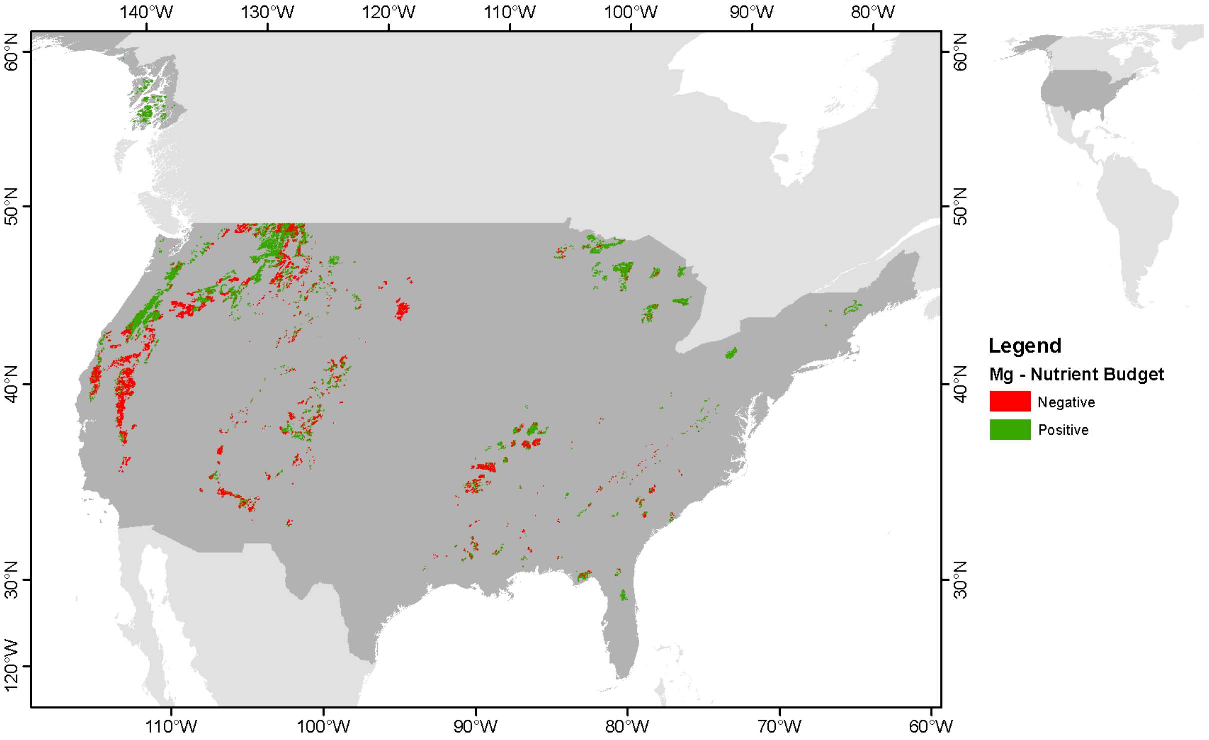

Figure S 22: U.S. timberland spatially-explicit nutrient budget for median atmospheric Mg deposition, and maximum (75th percentile) geogenic supply and harvest loss. Red colors indicate areas with higher harvest nutrient loss than nutrient supply. Green colors represent the opposite. For shown case, Mg nutrient deficiency occurs for 45% of timberland areas. Map generated with ESRI ArcGIS ver. 10.3.1 (http://www.esri.com).

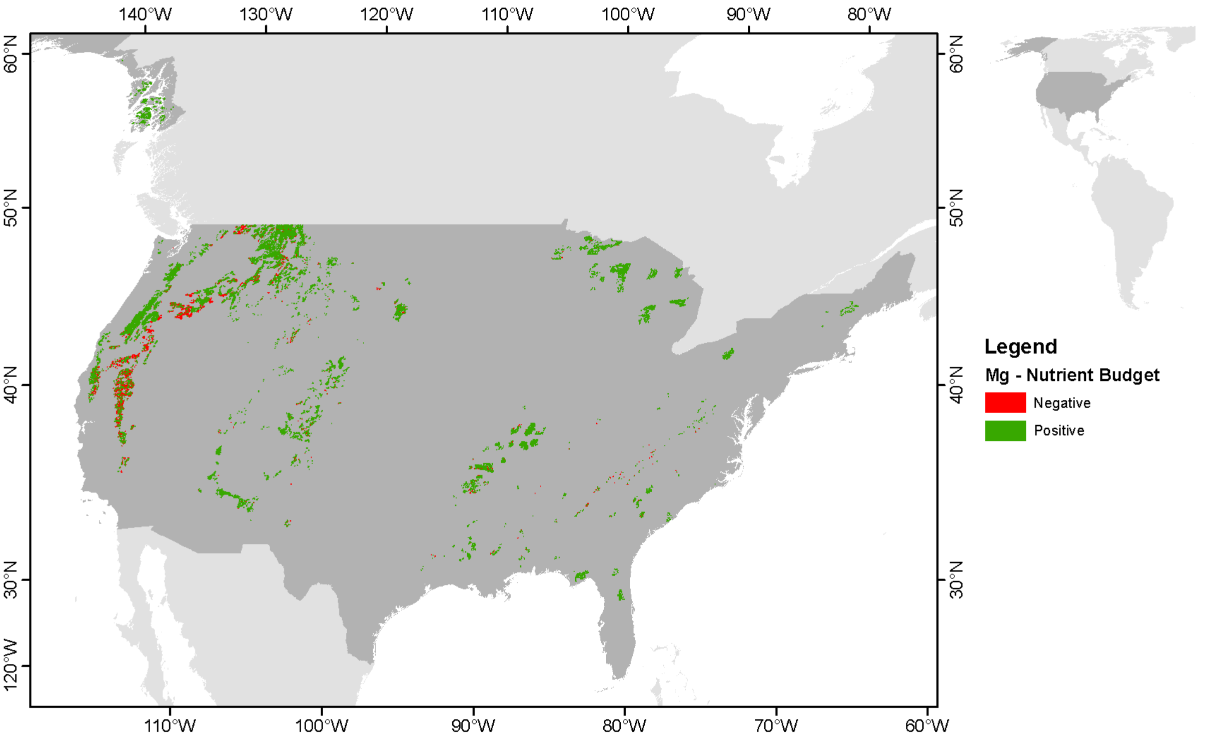

Figure S 23: U.S. timberland spatially-explicit nutrient budget for median atmospheric Mg deposition, and minimum (25th percentile) geogenic supply and harvest loss. Red colors indicate areas with higher harvest nutrient loss than nutrient supply. Green colors represent the opposite. For shown case, Mg nutrient deficiency occurs for 16% of timberland areas. Map generated with ESRI ArcGIS ver. 10.3.1 (http://www.esri.com).

Nutrient Budget for elements using two different scenarios

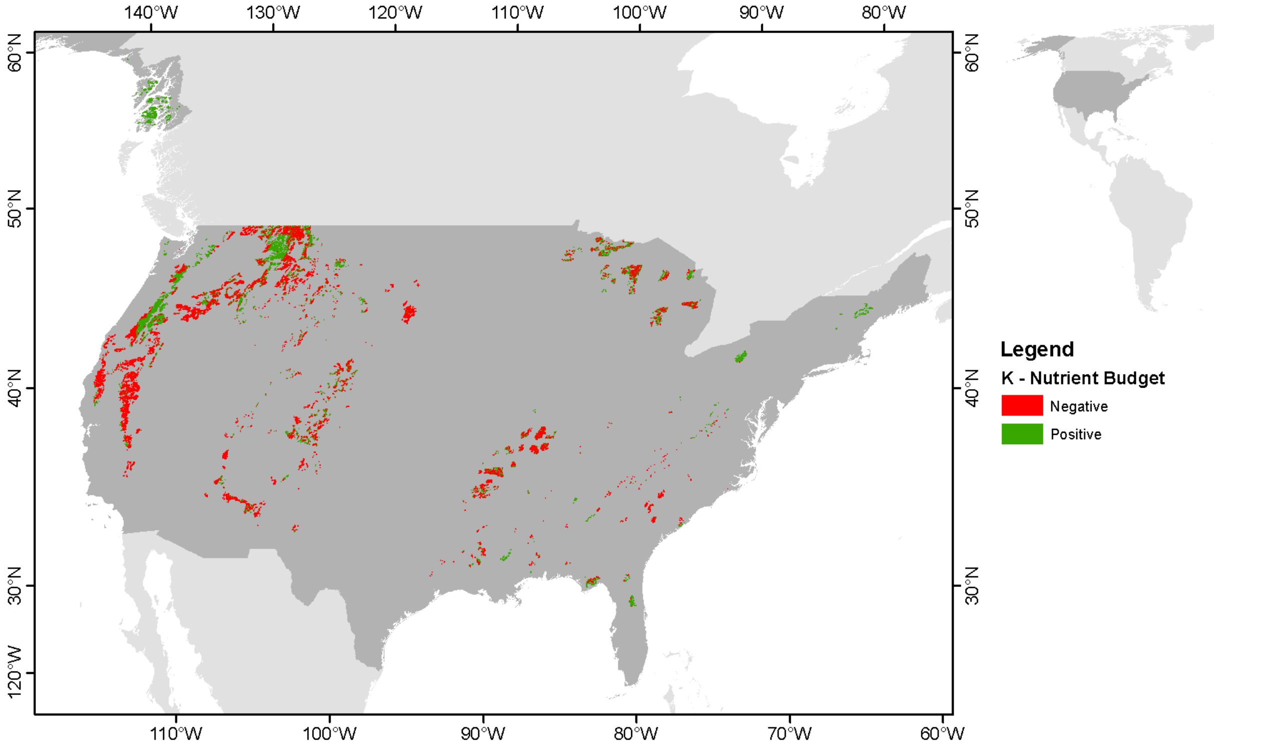

Figure S 24: U.S. timberland spatially-explicit nutrient budget for median atmospheric K deposition, and maximum (75th percentile) geogenic supply and harvest loss. Red colors indicate areas with higher harvest nutrient loss than nutrient supply. Green colors represent the opposite. For shown case, K nutrient deficiency occurs for 57% of timberland areas. Map generated with ESRI ArcGIS ver. 10.3.1 (http://www.esri.com).

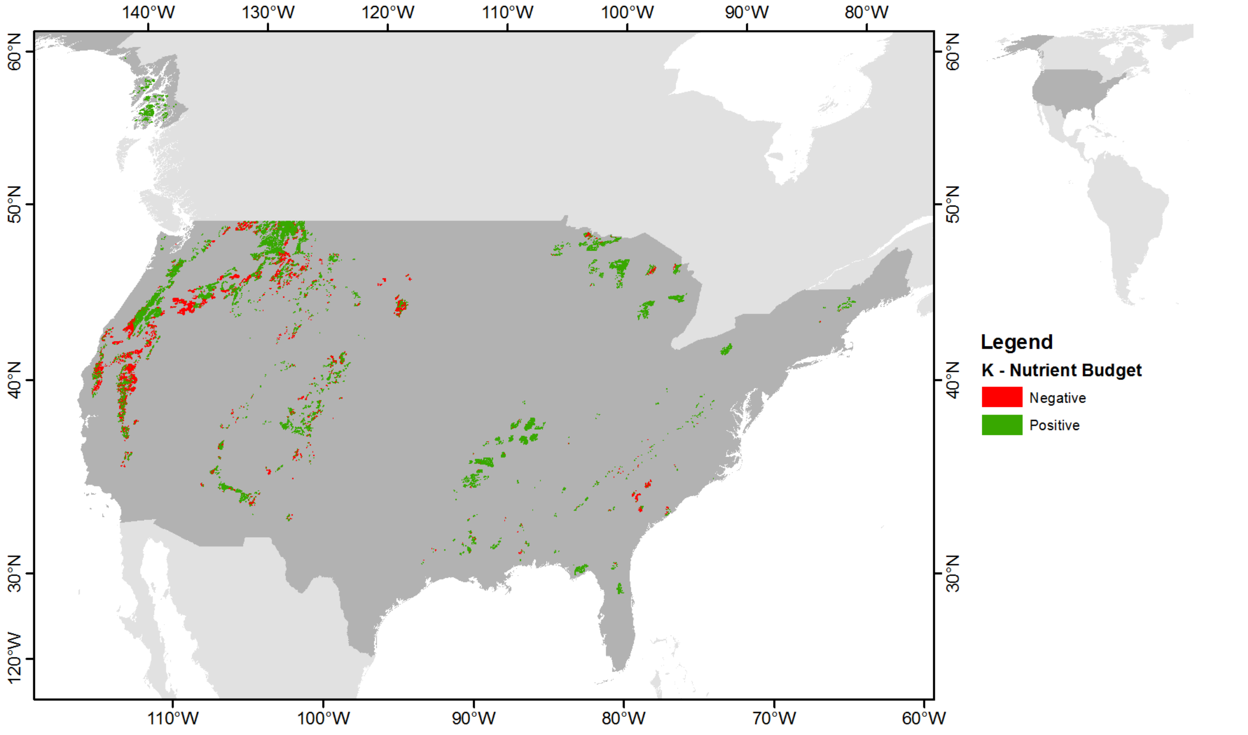

Figure S 25: U.S. timberland spatially-explicit nutrient budget for median atmospheric K deposition, and minimum (25th percentile) geogenic supply and harvest loss. Red colors indicate areas with higher harvest nutrient loss than nutrient supply. Green colors represent the opposite. For shown case, K nutrient deficiency occurs for 20% of timberland areas. Map generated with ESRI ArcGIS ver. 10.3.1 (http://www.esri.com).

D. References

1 USFS. *U.S. Forest Service - Timber Harvests*, <https://catalog.data.gov/dataset/u-s-forest-service-timber-harvests> (2016).

2 Survey, U. F. S. U. S. G. in *Forest Cover Types* (U.S. Geological Survey, Reston, VA, 2000).

3 Pardo, L. H. R.-A., M.; Duarte, N.; Miller, E., K. Tree Chemistry Database (Version 1.0). 45 (United States Department of Agriculture, NE-324. Newtown Square PA, 2005).

4 Laboratory, F. P. Wood handbook—Wood as an engineering material. Report No. General Technical Report FPL-GTR-190, 508 p. (U.S. Department of Agriculture, Forest Service, Forest Products Laboratory, 2010).

5 Hartmann, J., Moosdorf, N., Lauerwald, R., Hinderer, M. & West, A. J. Global chemical weathering and associated P-release - The role of lithology, temperature and soil properties. *Chemical Geology* **363**, 145-163, doi:10.1016/j.chemgeo.2013.10.025 (2014).

6 Program, N. A. D. *Total Deposition Maps, v2016.01*, <http://nadp.sws.uiuc.edu/committees/tdep/tdepmaps> (2016).

7 Schwede, D. B. & Lear, G. G. A novel hybrid approach for estimating total deposition in the United States. *Atmospheric Environment* **92**, 207-220 (2014).

8 Mahowald, N. *et al.* Global distribution of atmospheric phosphorus sources, concentrations and deposition rates, and anthropogenic impacts. *Global Biogeochemical Cycles* **22**, n/a-n/a, doi:10.1029/2008GB003240 (2008).

9 Sarbas, B. in *Geoinformatics 2008—Data to Knowledge* (eds Shailaja R. Brady, A. Krishna Sinha, & Linda C. Gundersen) 42 - 43 (USGS, Potsdam, 2008).

10 Hartmann, J. & Moosdorf, N. The new global lithological map database GLiM: A representation of rock properties at the Earth surface. *Geochemistry, Geophysics, Geosystems* **13**, n/a-n/a, doi:10.1029/2012GC004370 (2012).

11 Hartmann, J., Lauerwald, R. & Moosdorf, N. A brief overview of the GLObal RIver CHemistry Database, GLORICH. *Geochemistry of the Earth's Surface Ges-10* **10**, 23-27, doi:10.1016/j.proeps.2014.08.005 (2014).
